# Reducing growth and developmental problems in children: Development of an innovative postnatal risk assessment

**DOI:** 10.1371/journal.pone.0217261

**Published:** 2019-06-05

**Authors:** Minke R. C. van Minde, Lyne M. G. Blanchette, Hein Raat, Eric A. P. Steegers, Marlou L. A. de Kroon

**Affiliations:** 1 Department of Obstetrics & Gynecology, Division of Obstetrics and Prenatal Medicine, Erasmus University Medical Center, Rotterdam, The Netherlands; 2 Department of Public Health, Division Child & Youth, Erasmus University Medical Center, Rotterdam, The Netherlands; 3 Department of Social Development, Municipality of the city of Rotterdam, Rotterdam, The Netherlands; 4 University Medical Center Groningen, Department of Health Sciences, University of Groningen, Groningen, The Netherlands; Center for Healthy Start Initiative, NIGERIA

## Abstract

**Introduction:**

Globally, awareness of the relevance of both medical and non-medical risk factors influencing growth and development of children has been increasing. The aim of our study was to develop an innovative postnatal risk assessment to be used by the Preventive Child Healthcare (PCHC) to identify at an early stage children at risk for growth (catch-up growth, overweight and obesity) and developmental problems (such as motor, cognitive, psychosocial and language/ speech problems).

**Methods:**

We used the first four steps of the Intervention Mapping process. Step 1: Review of the literature and focus group discussions. Step 2: Identification of program objectives on how to develop and implement a risk assessment in PCHC daily practice. Step 3: Application of the ASE model to initiate behavioral change in the target group. Step 4: Development of the postnatal R4U and a program plan for the implementation in PCHC organizations.

**Results:**

Subsequently in 2015, the 41 item postnatal R4U (the postnatal Rotterdam Reproduction Risk Reduction checklist) was developed according to steps one until four of the Intervention Mapping process and was implemented in four PCHC organizations.

**Conclusions:**

It was feasible to design and implement a postnatal risk assessment identifying both medical and non-medical risks for growth and developmental problems, using the Intervention Mapping process.

## Introduction

The most common causes of perinatal morbidity are congenital anomalies, being born small for gestational age (SGA, birth weight under the 10^th^ percentile, adjusted for gestational age), preterm birth (before 37 weeks), or a low Apgar score (below 7 five minutes after birth) [1, 2]. The prevalence of perinatal morbidity is higher in deprived neighborhoods due to adverse effects of socio-economic non-medical risks [3–5]. The presence of both medical and non-medical risk factors predict adverse outcomes at birth [6] and influences long term health outcomes in children [7–9].

These children, for example born in a low socio-economic environment, have an increased risk of not reaching their developmental potential and of acquiring growth problems, such as obesity [10–13]. This vulnerability can persist into later life and can affect the health of their offspring, the next generation [14, 15]. Consequently, the accumulation of heterogeneous risk factors might be even more important than individual ‘high risk’ factors when it comes to adverse health outcomes [16–18].

Although Preventive Child Healthcare (PCHC) professionals seem to be aware of the importance of medical, as well as non-medical risk factors, such related risk assessment is currently not systematically applied, neither are related tailored care pathways. Our aim was to develop such a postnatal risk assessment, the postnatal Rotterdam Reproductive Risk Reduction checklist (postnatal R4U). With this instrument, PCHC professionals will be able to detect and weigh the severity of early medical and non-medical risk factors for growth and developmental problems in children. Subsequently, tailored care pathways can be offered to reduce these risks, in time.

In this study we aim to develop the postnatal R4U and tailored care pathways, as part of the Healthy Pregnancy 4 All-2 (HP4All-2) program [19]. HP4All-2 is the sequel of the HP4All program, initiated by the Erasmus Medical Center in cooperation with Dutch municipalities [3]. HP4All-2 aims to enforce and facilitate continuous care for families at risk after birth by focusing on antenatal and postnatal risk assessment in combination with tailored care pathways by maternity care, PCHC and interconception care [19].

## Materials & methods

### Trial registration

The qualitative study was reviewed by the Daily Board of the Medical Ethics Committee Erasmus MC as part of a larger study on implementation of interconception care in the Netherlands (MEC-2015-697). As a result of this review, the Board declared that the rules laid down in the Medical Research Involving Human Subjects Act (also known by its Dutch abbreviation WMO) do not apply to the study. No additional approval was requested for the current study since it is not based upon a clinical study or patient data.

### Development of the risk assessment

**Fig 1:**
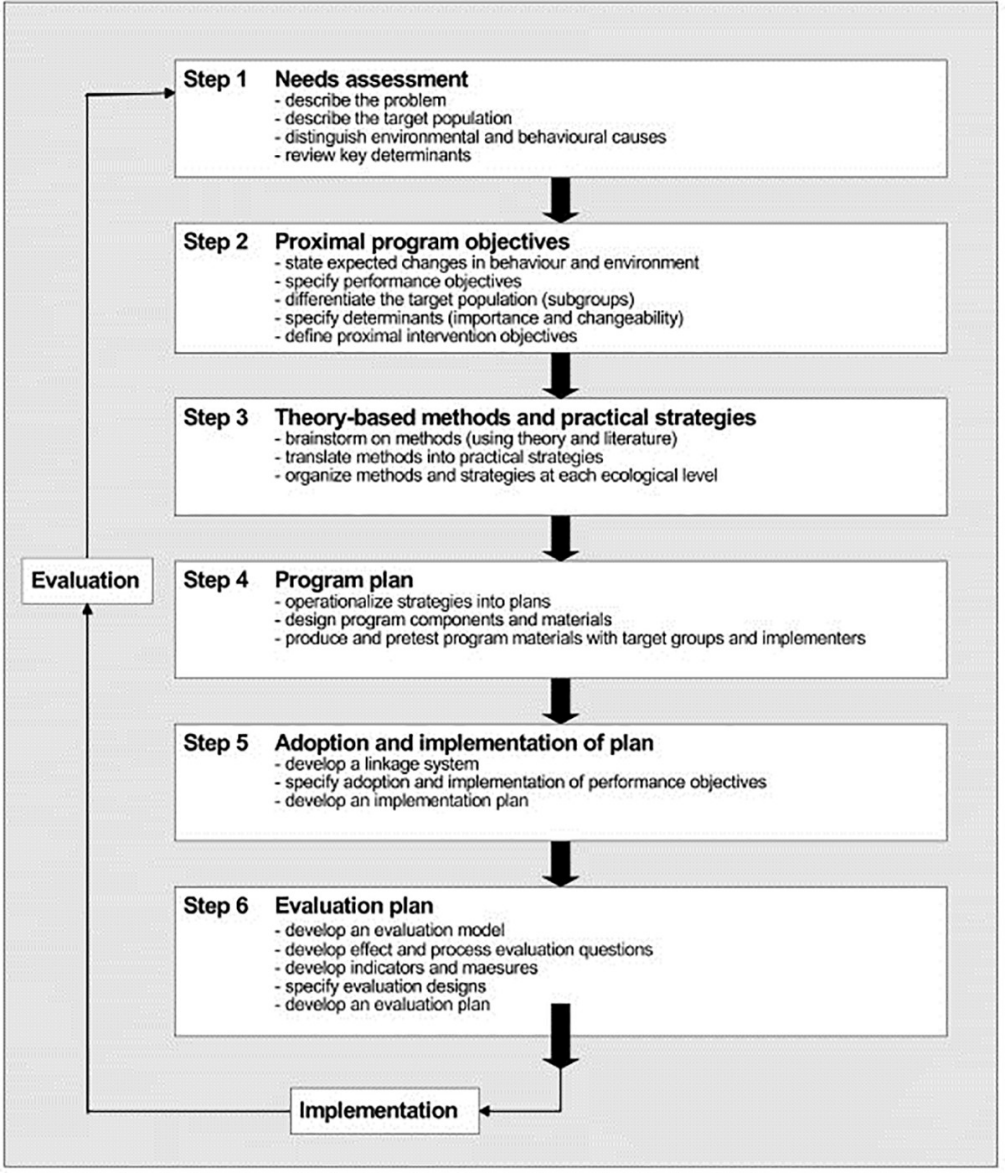
The six steps of the Intervention Mapping process [20].

In order to develop an innovative postnatal risk assessment, the postnatal R4U, in combination with tailored care pathways, the Intervention Mapping (IM) process was applied. IM is a protocol for the development of theory-based and evidence-based health promotion programs [20]. (Fig 1). In a recently published systematic review IM has been successfully used to plan, implement and evaluate interventions that showed a significant increase in uptake of disease prevention programs [21].

#### Step 1: Needs assessment

The IM process starts with a needs assessment of the health problem, which includes identification of risk factors, target groups, and of the aspired program outcomes. The methods used for the needs assessment were a study of the literature followed by three focus group discussions with relevant stakeholders.

Study of the literature. First, we performed an electronic literature search on the 12^th^ of February 2015 in Medline, Embase, Psycinfo and Cochrane for (1) risk factors of childhood overweight, obesity or catch-up growth, and (2) risk factors for developmental problems in children. Developmental problems were defined as psychomotor, cognitive, social and language/speech problems. Catch-up growth in early life has been associated in the literature with overweight, obesity and developmental problems in later life. [22–25] Attention was restricted to publications from western countries (because of generalizability to our target population) from 2005 onward, because of the amount of literature found in the search. We assumed that more recent publications would show the most relevant outcomes. A search strategy (2005–2015) was developed based on ‘perinatal risk factors’, ‘growth’ and ‘development’ and their Mesh terms. The search was restricted to Dutch and English.

Stakeholder consultations: focus group discussions. The second part of the needs assessment consisted of collecting information from important stakeholders. Therefore we organized three focus group discussions with stakeholders (with expertise on child growth and development and its risk factors), including physicians, nurses, researchers and policy makers from Obstetrics and Gynaecology, General Paediatrics and Neonatology, PCHC, Primary Healthcare and Research Institutions. This consultation enabled a deeper understanding of the context or communities in which the intervention was to be delivered [20]. During the discussions we addressed the nature of the problem and the findings of our literature review, seeking ideas on the presented associations and looking for risk factors that were missed in the literature. Additionally we discussed what the desired outcomes of the program should be, we identified both a primary and a secondary target group for the postnatal risk assessment (and corresponding care pathways) and discussed how the program should be implemented within the PCHC organizations. One of the researchers moderated the discussions, another researcher took notes. The three focus group discussions were tape-recorded with verbal informed consent from the participants and were subsequently transcribed. Data were analysed using the program Nvivo (version 11.4.1/February 2017, Qualitative Data Analysis computer software package, QSR International software), for qualitative data analysis. To integrate results of the discussions and literature review, themes derived from the discussions were linked to risk factors found in literature.

#### Step 2: Specification of proximal program objectives

**Fig 2:**
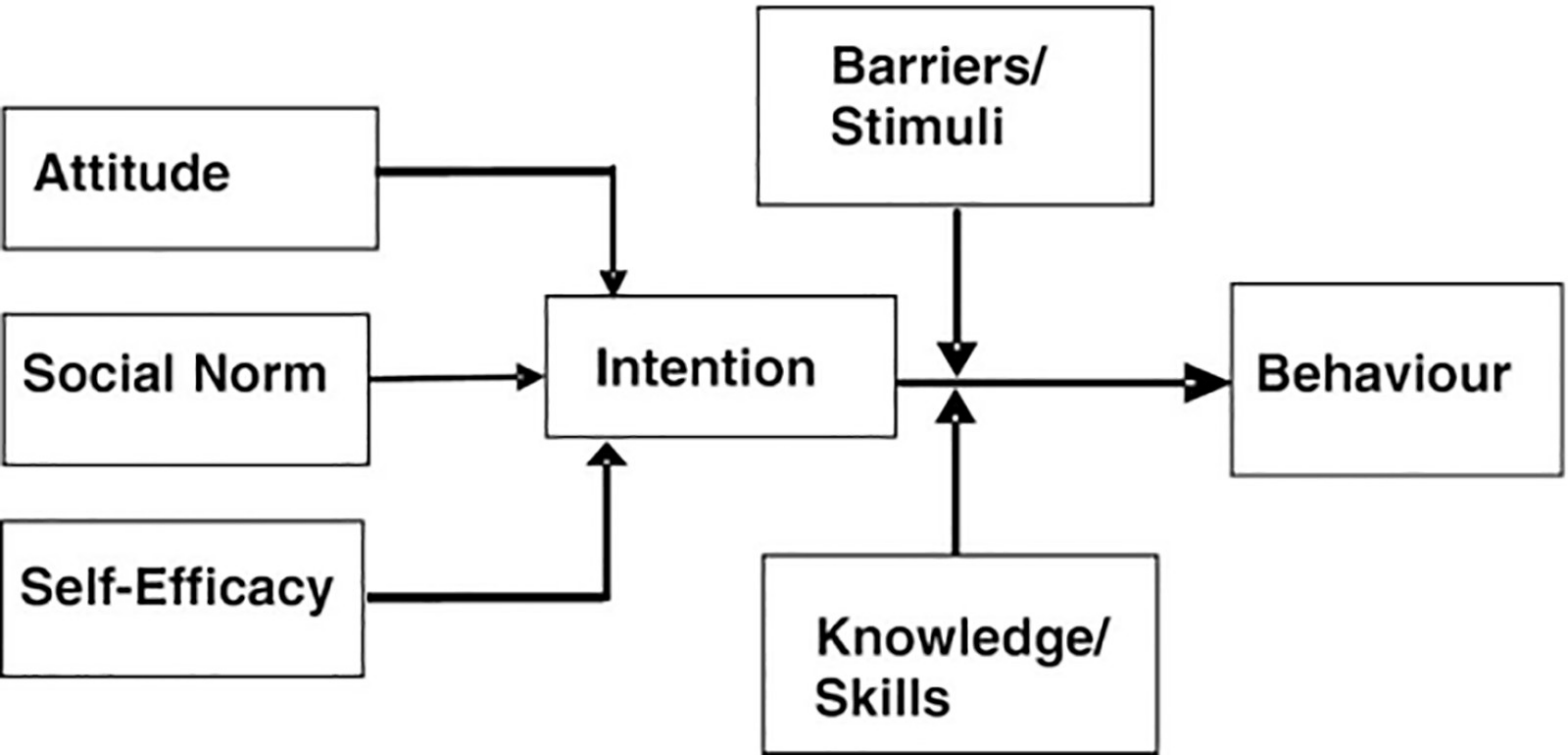
The ASE model which was applied to accomplish behavioral change in PCHC professionals.

The objectives of the program were specified in step two of the IM process [20]. Based on the program outcomes formulated in the needs assessment, different performance objectives were conveyed at the individual level (PCHC nurses and physicians) and at the interpersonal level (PCHC organizations). These performance objectives stated what the involved professionals had to do or how the PCHC organizational environment had to be modified in order to successfully introduce and implement the postnatal R4U, and thereby contribute to optimal health related outcomes and preventing growth and developmental problems in children. The literature review and the focus group discussions supported us to identify important behavioral and environmental determinants of behavior change of professionals. Subsequently, we identified a suitable theoretical model, referred to as the attitude/ social norm/ self-efficacy model (ASE model) [26] as the most applicable model to use as a basis for the development of the implementation process of the postnatal R4U and related care pathways. ASE is a model that has general scientific acceptance and explains behavior by linking various determinants such as attitude, social norm and and self-efficacy with behavioral intention and behavior [27]. Therefore, this model seemed to be appropriate as a basis to guide the way in which the PCHC professionals can be involved and ensure a permanent behavioral change within their daily practices. Fig 2 shows the process of this model.

#### Step 3: Theoretical model, theory-based methods and practical strategies

In the third step of the IM process we focused on different methods of change.

In the ASE model, it is assumed that intention and subsequent behavior are primarily determined by the following cognitive variables: attitudes, social influences/norms, and self-efficacy expectations. Moreover, the model postulates that intention predicts subsequent behavior. A person’s attitude towards a specific behavior (e.g. applying a new risk assessment instrument in daily practice) is a result of the consequences that a person expects from performing the behavior (e.g. “applying this risk assessment instrument will take extra time during my consultation”). Social influences can be described as the processes whereby people directly or indirectly influence the thoughts, feelings, and actions of others. Self-efficacy expectations pertain to a person’s belief in his or her ability to perform desired behavior [28].

Subsequently, appropriate theoretical methods could be selected and translated into practical strategies in order to positively influence each of the identified determinants. Related materials and tools were developed. Taken together, these elements would ensure ongoing implementation and a persistent behavioral change of the involved professional’s and their organization.

#### Step 4. Producing intervention components and materials

During the fourth step of the IM process, the information from all previous steps was combined and led to the development of the postnatal R4U, related care pathways and different program components and materials. Most of the program components and materials were tested and revised based on feedback from PCHC professionals in the participating municipalities.

#### Step 5. Planning program adoption and implementation

During the fifth step of the IM process, the intervention adoption and implementation was planned. Based on the first step of the IM process, the PCHC physicians and nurses were considered as the intervention adopters and implementers. Demographic and cultural differences of the target population at the specific PCHC locations were taken into account to ensure feasibility of implementing the postnatal R4U and its corresponding care pathways in all participating PCHC organizations. Subsequently, a clear implementation plan and training was developed to inform all PCHC employees in the participating organizations about the different core components of the intervention and about details on how to deliver the intervention to the parents visiting PCHC. The emphasis of the implementation plan was placed on achieving a high level of commitment and completeness. During the implementation plan we focused on flexibility and easy to use methods to ensure and easy adoption.

#### Step 6. Evaluation planning

The last step of the IM process included the development of a plan for the evaluation of the outcomes and the process of the intervention. A process evaluation as well as an outcome and efficacy evaluation were planned.

## Results

### Results of step 1: Needs assessment

#### Study of the literature

The full literature search can be found in S1 File. The initial literature search resulted in 12,039 articles. After excluding the articles published before 2005, 7049 articles remained. After screening the titles and abstracts on eligibility, 496 were left for full article reading. These articles were structurally reviewed for the following topics: predicted outcome (growth and/or development), identified risk factor(s) from preconception until the age of 8 weeks old, type of analysis and statistical results (e.g. odds ratio, hazard ratio, risk ratio, regression coefficients), quality of the study, age of the children during the assessment, possible confounders, generalizability and size of the research cohort. These papers were read by the first reviewer. 376 articles were excluded because they lacked statistical results, did not assess the predicted outcome or did not assess relevant risk factors. 120 articles remained for a second reviewer within the project team and were scored on generalizability, validity and overall quality, by at least two reviewers. Finally, 69 articles remained to be considered, according to their high scores. Additionally, several articles suggested by participants in the focus groups were added and reviewed. 9 articles were approved according to the above mentioned criteria by two reviewers. This resulted in 78 articles that were eligible for the risk assessment. These articles described a wide range of risk factors influencing growth and development of children, each article containing one or more risk factors. Social risk factors included low socio-economic status and ethnicity. Maternal risk factors included maternal psychological/psychiatric problems, intoxications such as smoking and drug abuse, gestational diabetes and maternal overweight. Fetal/neonatal risk factors included small and large for gestational age, preterm birth and a low Apgar score. See S2 File for the full list of included articles and the identified risk factors.

#### Focus group discussions

The three focus group discussions each respectively included 8, 9 and 15 stakeholders, with a median age of 45 years old (range 25–65 years old), of which 90% was female. The discussions lasted between 140 and 150 minutes, with a mean of 145 minutes.

We identified a need for an early, systematic and evidence based postnatal risk assessment within PCHC, in which the accumulation of risk factors can be taken into account and care pathways can be selected. Indeed, during the focus group discussions the participants stressed on the fact that a risk assessment can not exist without corresponding care pathways. Identifying a risk should lead towards suitable care to prevent further risk or negative outcomes in the future. As a result, we decided to organize a third focus group discussion concerning the development of tailored care pathways.

During the focus group discussions we identified the PCHC physicians and nurses as the primary target group of the intervention. The secondary target group consists of their clients; parents and their children from 0 until 8 weeks old.

The age from 0 until 8 weeks old was chosen because the assessment has to take place in the early postnatal period. A maximum of 8 weeks was chosen because of organizational reasons; the home visit by the PCHC nurse takes place between 12–14 days after birth, during which many items of the postnatal R4U are discussed according to protocol. At 4 weeks a consultation by the PCHC physician is scheduled, and at 8 weeks another consultation by the PCHC nurse takes place at the specific PCHC location. During this consultation extra focus can be given to the social domain, in which certain items included in the postnatal R4U can be addressed as well. In order to ensure that the R4U can be implemented during standard care, without putting too much weight on one single visit, we chose these three eligible consultations for the risk assessment using the postnatal R4U. In case of preterm birth, the corrected age can be applied, to safeguard referral to appropriate care and participation in the study.

Results from the focus group discussions are presented in S2 File.

#### Aspired program outcomes

Based upon the above mentioned results, specific aspired outcomes were formulated in order to evaluate the effectivity of the program.

Primary outcomes are overweight (>1 SDS for length), obesity (>2 SDS for length) [29] and catch-up growth (>0,67 SDS) [30] and developmental problems (psychomotor, cognitive, psychosocial and language/speech) in the first six months of life. Secondary outcomes are the use of the postnatal R4U and its corresponding care pathways by PCHC professionals and their knowledge, attitude and intention after the implementation.

#### Results of step 2: Proximal program objectives

The selection of the risk factors to be included in the postnatal R4U was carefully discussed within the research team, with regards to scientific evidence as well as the implementation feasibility in PCHC organizations. Hence, certain risk factors were not selected due to lack of evidence (i.e. pets in the household influencing child development, which was only mentioned in one article) or the infeasibility of applying it in PCHC daily practice (such as hemoglobin levels of the mother, which can not be measured in PCHC).

Two main program objectives were identified on how to develop and implement a risk assessment in PCHC daily practice. First, risk factors should be identified in a systematic manner by the healthcare professionals, in order to be able to screen objectively and without missing any risks. Second, based on the risk assessment, care pathways should be identified and developed, assisting professionals to direct parents to the appropriate care within a certain neighborhood or municipality. The identified important behavioral and environmental determinants of behavior change of the professionals were attitude, social influence, self-efficacy and PCHC organizational environment.

#### Results of step 3: Theoretical model, theory-based methods and practical strategies

The ASE theoretical model [31] enabled us to consider all different determinants of professional behavior and the way these determinants interact together and might influence a person’s intention and subsequent behavior. From there on, we were able to select appropriate theoretical methods and conceptualize practical strategies and tools for the implementation of the postnatal R4U and care pathways (see Table 1).

**Table 1:** Personal and environmental determinants according to the ASE model, theoretical methods, preconditions, practical strategies and tools for the design and implementation of the postnatal risk assessment and care pathways (Rotterdam, The Netherlands, 2015).

Determinant	Theoretical method	Precondition	Practical strategy	Tools	Who is responsible
**Attitude**	Passive/active learning	Credibility and clarity of the source. Knowledge of trainer and/or teacher	Research group and management of the organization provide emphatic, accessible written and verbal information.	Group training on scientific evidence of risk factors influencing child growth and development. /Provision of all background information including all identified risk factors, care pathways and literature references. / Pocket size guide for the use of the postnatal R4U and its corresponding care pathways.	Research team including a professional communication specialist. /Research team./Research team.
**Social influence/norm**	Mobilizing social support and control	Involvement of management and staff of the organization. /Involvement and presence of research group.	Management and staff: monitor, encourage and remind professionals. /Research group: updates on the statistics of the risk assessment.	Discuss the progress of the program during team consultations, sent frequent reminders about the risk assessment. /Frequent visits to the PCHC locations and presentations on the statistics of the study.	Research team and PCHC management. /Research team.
**Self-efficacy**	Passive/active and interactive learning	Credibility and clarity of the source. /Knowledge of trainer and/or teacher.	Research group and management of the organization provide emphatic, accessible written and verbal information.	Group training by professional trainer on communication strategies in case of parents who are in resistance. /Syllabus/hand out on communication models.	Research team and professional communication specialist./Research team.
**Environment**	Environmental changes	Involvement of all PCHC professionals.	Management and staff: provide secure environment in which there is time and space to implement and work with the postnatal R4U. /ICT: Adjustment of the digital file to facilitate working with the postnatal R4U	Prolonged consultations. /Postnatal R4U embedded in digital file.	PCHC management. / Research team and ICT.

#### Results of step 4: Producing intervention components and materials

**Fig 3:**
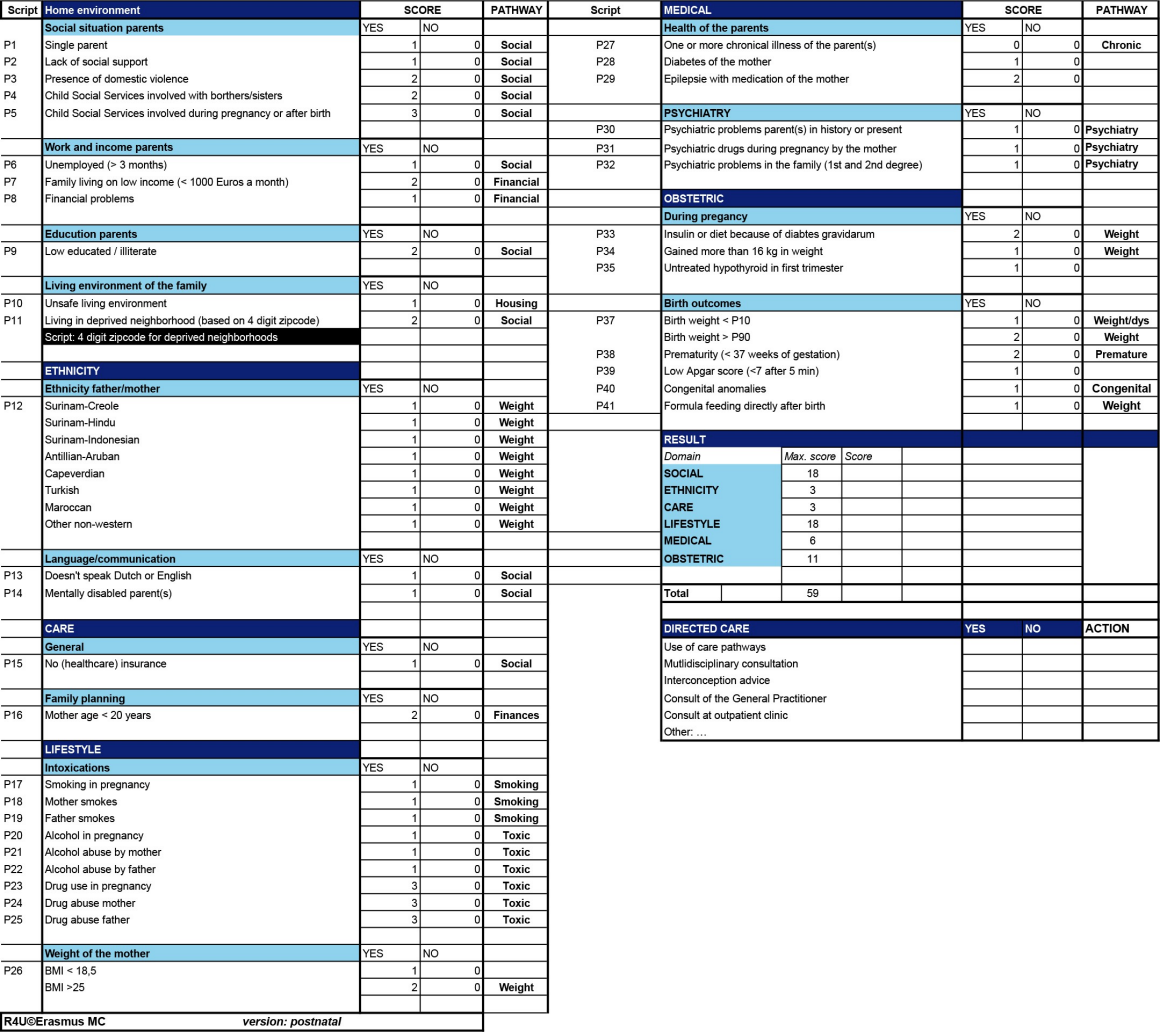
Postnatal R4U: Presenting risk factors (second column), the corresponding score (column 3 and 4) and care pathway(s) (column 5).

The postnatal R4U. The postnatal R4U was created using the previous mentioned steps in the IM process. See Fig 3 for the result.

Risk factors identified in the literature or the focus group discussions were categorized into different domains: (1) social, (2) ethnic descent and language barriers, (3) lifestyle, (4) healthcare behaviors, (5) general medical and (6) obstetric.

During the development we used ‘weighing’ and a cumulative risk score, as has been done in a precedent study [32]. To obtain a cumulative risk score for an individual patient, weights have to be assigned to each positive item. A cumulative risk score above a predefined threshold would imply the need for a multidisciplinary consultation between PCHC professionals and other healthcare providers. The authors propose a threshold of 15, since this would imply a large amount of different accumulating risk factors for a patient or family. This offers the opportunity to customize care policy to the specific needs of a child and his/her family. Such a threshold may be locally adapted to accommodate the availability of facilities [32]. For instance when the workload of the multidisciplinary consultations is too scarce or too heavy or when professionals feel that certain vulnerable families with a lower score than 15 should benefit from a multidisciplinary consultation. These specific organizational needs could be subject to environmental factors in a certain neighborhood or municipality. After the implementation study, it might be possible to define a more evidence based threshold, using statistical analysis.

We expressed weights in points, depending on odds ratios/relative risks mentioned in the reviewed literature (S3 File). Risk factors consistently associated with odds ratios/relative risks smaller than two were assigned 1 point, higher than two were assigned 2 points, and for risk factors associated with odds ratios/relative risks higher than four, 3 points were assigned. For some items, expert opinion prevailed, due to missing odds ratios/relative risks in the literature, such as substance abuse of a parent. The item chronic illness of the parent, for which there was no evidence in the literature review, received 0 points.

Care pathways. Tailored care pathways were developed in collaboration with PCHC professionals themselves, including staff members, physicians and nurses and with other local healthcare providers, such as social welfare workers. Care pathways were: ‘psychosocial’, ‘financial and housing problems’, ‘weight’, ‘smoking’, ‘substance abuse’, ‘chronic illness’, ‘psychiatry’, ‘preterm birth/SGA’ and ‘congenital anomalies’. Each care pathway was individually designed for a participating municipality or neighborhood. These care pathways are very elaborate and specifically designed for one of the participating PCHC organizations. Therefore, we added examples of the care pathways developed. See S3 File.

#### Results of step 5: planning program adoption and implementation

An implementation plan was designed. As a result, a group training was developed, in collaboration with a professional training company, to inform and educate PCHC professionals on the postnatal R4U, its scientifically identified risk factors and its corresponding care pathways. Program materials were developed and distributed, such as posters for the PCHC organizations, flyers for the parents and educational booklets for the PCHC professionals.

The postnatal R4U has been incorporated in the digitized files of the PCHC centers, automatically transferring data of already obtained relevant risk factors from the digital file to the risk assessment.

#### Results of step 6: Evaluation planning

As the last step of the IM process, a process evaluation of the pilot implementation, using a questionnaire for PCHC professionals and a meeting for the evaluation of the intervention with PCHC professionals, PCHC management and municipality officials will take place. The outcome and efficacy of the postnatal R4U will be analyzed using an intervention cohort (n = 3120), in which the postnatal R4U has been implemented, and an historical cohort (n = 3120), in which the instrument has not been used. Child growth and development in both cohorts will be compared. The design of this specific study will be published separately.

## Discussion

We have developed a postnatal risk assessment for PCHC organizations, using the steps of the IM process.

The IM process is one of many validated methods for intervention development in medical sciences. In the past years, IM has been successfully used to plan, implement and evaluate interventions that showed a significant increase in uptake of disease prevention programs [21]. Another method, primarily used in pediatric psychology, is an author’s checklist for measure development by Holmbeck and Devine (2009) [33]. Similar to IM they highlight the establishment of the scientific need for the instrument as well as clinical experience, rational deduction, related instruments and consultation with experts. Unlike IM they focus on the evaluation of the diagnostic utility and translating the measure in other languages. Because IM was more broadly used in the medical field, we chose this method.

This instrument, the postnatal R4U, enables to screen for medical and non-medical risk factors that influence a child’s growth and development. This assessment can be done in a structured manner, at an early age, and subsequently, offer care using the corresponding care pathways.

As most children and their parents visit the PCHC locations on a regular basis, PCHC plays an important role in the primary prevention of these problems by timely identification and advising parents or referring them to other care providers [34]. Therefore, an instrument for a swift and structured identification of risk factors accompanied by corresponding care pathways seems valuable.

Within the HP4All program, an antenatal risk assessment (the antenatal R4U) has been developed to assess in early pregnancy, the risks of congenital anomalies; being born small for gestational age; preterm birth; and a low Apgar score. The antenatal R4U has been evaluated in multiple research projects [6, 32, 35]. The postnatal R4U seems a good sequel of the antenatal R4U in order to screen for the antenatal R4U main outcomes and other risk factors [36], which can separately increase the risk of growth and developmental problems in affected children [22, 37, 38].

In different fields of preventive healthcare and pediatrics, risk assessments have been developed, such as a psychosocial risk assessments [39] and the child abuse inventory at emergency rooms [40, 41]. In Child Preventive Healthcare the SPARK, an instrument for the early detection of developmental problems in toddlers has been recently developed [42, 43]. A postnatal risk assessment, which screens for both medical and non-medical risk factors, has, to our knowledge, not previously been developed.

In the future, after demonstrating the effectiveness and efficacy of the postnatal R4U, based on the evaluation study, national implementation of the postnatal R4U may be advised. The Dutch Center of Youth Health (www.ncj.nl), which is nationally responsible for promoting the implementation of guidelines and new working methods in PCHC, would have an important role in the national implementation process of the postnatal R4U. Moreover, as of November 2018 the postnatal R4U has been made available on a digitized promotional forum for PCHC organizations, where they can view and pilot possible digital instruments for their organization.

### Strengths and limitations

When developing the risk assessment, we performed a literature search on the most recent published data on perinatal risk factors and their influence on growth and development of children. By doing so, we tried to gain knowledge on the background of the problem, as suggested by Moore et al [44]. This resulted in scientifically identified risk factors influencing child growth and development. Moore also underlines the importance of consulting important stakeholders, while developing a new intervention, through intervention coproduction [44]. In order to consider the point of view of professionals during the development and implementation of the postnatal R4U, we involved important stakeholders in Preventive Child Healthcare during the focus group interviews, which we consider a strength of this study. However, during this process, we did not consider the opinion of the parents, the clients of PCHC. We chose not to do so because our aim was to facilitate current care practices in PCHC and first target the attitude, intention and behavior of the caregivers. Nevertheless, in future research the opinion of parents concerning the offered care to their children should be considered.

Although many factors influencing the health and the wellbeing of children have been studied, research on the influence of non-medical risk factors on child development remains scarce. In this study, we tried to overcome this issue by consulting stakeholders in focus group discussions in addition to the literature review.

A postnatal risk assessment for child growth and development is probably most effective if corresponding care pathways direct to the appropriate care. These care pathways should be known to the professionals and time should be allocated to study them properly. In addition, the subsequent care offered should be accessible for parents and children. We aim to evaluate the accessibility to these care pathways, together with studying the predictive value of the postnatal R4U, in an implementation study.

## Conclusions

In conclusion, we successfully designed a postnatal risk assessment, the postnatal R4U, and related care pathways using the Intervention Mapping process. Moreover, we were able to implement the postnatal R4U, which is currently being used and evaluated in four PCHC organizations. Future research will involve the evaluation of the assessment and will show whether such early risk identification and related care pathways may result in a decrease of growth and developmental problems in children.

## Supporting information

S1 File Literature search. (DOCX)

S2 File Supplementary tables. (DOCX)

S3 File Care pathways. (DOCX)

S4 File Question route focus groups. (DOCX)

S5 File PRISMA 2009 checklist. (PDF)

## Abbreviations

ASE model: attitude/ social norm/ self-efficacy model
HP4All-2: Healthy Pregnancy 4 All-2
IM: Intervention Mapping
PCHC: Preventive Child Healthcare
R4U: Rotterdam Reproductive Risk Reduction checklist
SGA: small for gestational age
