# Correction: Reducing growth and developmental problems in children: Development of an innovative postnatal risk assessment

**DOI:** 10.1371/journal.pone.0224160

**Published:** 2019-10-15

**Authors:** Minke R. C. van Minde, Lyne M. G. Blanchette, Hein Raat, Eric A. P. Steegers, Marlou L. A. de Kroon

There are multiple errors in the Abstract:

- The first and fifth sentences of the Methods subsection of the Abstract are incorrect.
- The first sentence of the Results subsection of the Abstract is incorrect.
- Two sentences are missing following the fifth sentence of the Methods subsection of the Abstract.

Please view the full, correct Abstract here.

## Abstract

### Introduction

Globally, awareness of the relevance of both medical and non-medical risk factors influencing growth and development of children has been increasing. The aim of our study was to develop an innovative postnatal risk assessment to be used by the Preventive Child Healthcare (PCHC) to identify at an early stage children at risk for growth (catch-up growth, overweight and obesity) and developmental problems (such as motor, cognitive, psychosocial and language/ speech problems).

### Methods

We used the Intervention Mapping process. Step 1: Review of the literature and focus group discussions. Step 2: Identification of program objectives on how to develop and implement a risk assessment in PCHC daily practice. Step 3: Application of the ASE model to initiate behavioral change in the target group. Step 4: Development of the postnatal R4U and corresponding care pathways. Step 5: Design of the program adoption and implementation in four PCHC organizations. Step 6: Planning program evaluation by a questionnaire and an evaluation meeting.

### Results

Subsequently in 2015, the 41 item postnatal R4U (the postnatal Rotterdam Reproduction Risk Reduction checklist) was developed according to steps one until six of the Intervention Mapping process and was implemented in four PCHC organizations.

### Conclusions

It was feasible to design and implement a postnatal risk assessment identifying both medical and non-medical risks for growth and developmental problems, using the Intervention Mapping process.

## References

[1] van Minde MRC, Blanchette LMG, Raat H, Steegers EAP, Kroon MLAd (2019) Reducing growth and developmental problems in children: Development of an innovative postnatal risk assessment. PLoS ONE 14(6): e0217261 10.1371/journal.pone.0217261 31166964 PMC6550373

