# Supplementary material for: Reducing growth and developmental problems in children: Development of an innovative postnatal risk assessment
Source: PLoS One. 2019 Jun 5;14(6):e0217261. doi: 10.1371/journal.pone.0217261 (PMC6550373; doi:10.1371/journal.pone.0217261)
Supplement: S1 File — (DOCX) [file pone.0217261.s001.docx]

**S1 File. Literature search**

| Embase.com | 9106 | 9017 |
| --- | --- | --- |
| Medline (OvidSP) | 7275 | 2236 |
| Psycinfo (OvidSP) | 1480 | 654 |
| Cochrane | 547 | 132 |
| **Total** | **18408** | **12039** |

**Embase.com 9106**

('growth disorder'/de OR 'failure to thrive'/de OR 'growth retardation'/de OR 'postnatal growth'/exp OR 'postnatal development'/exp OR 'growth curve'/de OR 'short stature'/de OR stunting/de OR 'body height'/de OR 'body weight disorder'/de OR obesity/de OR 'childhood obesity'/de OR 'body mass'/de OR 'developmental disorder'/de OR 'psychomotor development'/de OR 'motor development'/de OR 'language development'/de OR 'developmental language disorder'/exp OR 'speech development'/de OR 'psychosocial development'/de OR 'child development'/de OR 'head circumference'/exp OR 'body composition'/exp OR development/de OR (((behav*) NEAR/3 (disorder* OR disturba* OR anomal* OR arrest* OR failure* OR retard* OR deficien* OR stunt* OR disabilit* OR abnormal* OR restrict* OR problem* OR outcome* OR impair*)) OR (failure* NEAR/3 thrive*) OR underdevelop* OR growth OR development* OR ((short* OR small) NEAR/3 stature*) OR stunting OR (body NEAR/3 (height* OR length* OR stature OR small OR weight OR mass* OR fat OR composition)) OR obes* OR overweight* OR adipos* OR bmi OR ((psychomot* OR motor* OR language* OR speech* OR psychosocial OR psycho-social OR psychologic*) NEAR/3 (develop* OR delay*)) OR ((development* OR growth) NEAR/3 delay*) OR neurodevelopment* OR ((child* OR infan*) NEAR/3 development*) OR (grow* NEAR/3 curve*) OR ((head OR cranial) NEAR/3 circumference)):ab,ti) AND ('newborn assessment'/de OR 'newborn screening'/de OR 'developmental screening'/exp OR 'risk factor'/de OR 'risk assessment'/exp OR 'high risk population'/de OR screening/de OR prediction/exp OR Prognosis/exp OR 'scoring system'/de OR 'rating scale'/de OR ((risk NEAR/3 (factor* OR high* OR assess*)) OR screen* OR predict* OR scoring OR rating OR scale*):ab,ti) AND ('newborn period'/de OR newborn/de OR 'perinatal period'/de OR 'puerperal disorder'/exp OR (baby OR babies OR newborn* OR neonat* OR (birth* NEAR/3 cohort*) OR postnatal* OR puerper* OR perinatal*):ab,ti) AND (infant/de OR infancy/de OR (infan* OR ((2 OR two OR 1 OR one OR first OR second) NEXT/2 year*)):ab,ti) AND ('cohort analysis'/exp OR 'longitudinal study'/exp OR 'retrospective study'/exp OR 'prospective study'/exp OR 'controlled study'/exp OR 'follow up'/exp OR (cohort* OR longitudinal* OR retrospectiv* OR prospectiv* OR control* OR trial* OR 'follow up'):ab,ti) AND [english]/lim NOT ([Conference Abstract]/lim OR [Letter]/lim OR [Note]/lim OR [Conference Paper]/lim OR [Editorial]/lim OR 'case report'/exp OR 'case report':ti) NOT ('congenital deafness'/de OR hearing/exp OR 'hearing disorder'/exp OR Africa/exp OR Asia/exp OR 'developing country'/exp OR (deaf* OR hearing* OR Africa OR Asia OR ((developing OR underdevelop*) NEAR/3 countr*)):ab,ti) NOT ([animals]/lim NOT [humans]/lim)

**Medline (OvidSP) 7275**

(Growth Disorders/ OR failure to thrive/ OR "Growth and Development"/ OR "Growth and Development".xs. OR Growth Charts/ OR Dwarfism/ OR body height/ OR obesity/ OR Pediatric Obesity/ OR Body Mass Index/ OR Developmental Disabilities/ OR Child Development/ OR language development/ OR Language Development Disorders/ OR exp body composition/ OR (((behav*) ADJ3 (disorder* OR disturba* OR anomal* OR arrest* OR failure* OR retard* OR deficien* OR stunt* OR disabilit* OR abnormal* OR restrict* OR problem* OR outcome* OR impair*)) OR (failure* ADJ3 thrive*) OR underdevelop* OR growth OR development* OR ((short* OR small) ADJ3 stature*) OR stunting OR (body ADJ3 (height* OR length* OR stature OR small OR weight OR mass* OR fat OR composition)) OR obes* OR overweight* OR adipos* OR bmi OR ((psychomot* OR motor* OR language* OR speech* OR psychosocial OR psycho-social OR psychologic*) ADJ3 (develop* OR delay*)) OR ((development* OR growth) ADJ3 delay*) OR neurodevelopment* OR ((child* OR infan*) ADJ3 development*) OR (grow* ADJ3 curve*) OR ((head OR cranial) ADJ3 circumference)).ab,ti.) AND (Neonatal Screening/ OR risk factors/ OR risk assessment/ OR prediction/ OR prognosis/ OR ((risk ADJ3 (factor* OR high* OR assess*)) OR screen* OR predict* OR scoring OR rating OR scale*).ab,ti.) AND (Puerperal Disorders/ OR (baby OR babies OR newborn* OR neonat* OR (birth* ADJ3 cohort*) OR postnatal* OR puerper* OR perinatal*).ab,ti.) AND (infant/ OR (infan* OR (("2" OR two OR "1" OR one OR first OR second) ADJ2 year*)).ab,ti.) AND (exp Cohort Studies/ OR exp Clinical Trial/ OR Follow-Up Studies/ OR (cohort* OR longitudinal* OR retrospectiv* OR prospectiv* OR control* OR trial* OR follow up).ab,ti.) AND english.la. NOT (letter OR news OR comment OR editorial OR congresses OR abstracts).pt. NOT (deafness/ OR exp hearing/ OR exp Hearing Disorders/ OR exp Africa/ OR exp Asia/ OR developing countries/ OR (deaf* OR hearing* OR Africa OR Asia OR ((developing OR underdevelop*) ADJ3 countr*)).ab,ti.) NOT (exp animals/ NOT humans/)

**Psycinfo (OvidSP) 1480**

(failure to thrive/ OR exp body height/ OR exp obesity/ OR Body Mass Index/ OR exp Developmental Disabilities/ OR exp Childhood Development/ OR exp Infant Development/ OR exp Cognitive Development/ OR exp language development/ OR (((behav*) ADJ3 (disorder* OR disturba* OR anomal* OR arrest* OR failure* OR retard* OR deficien* OR stunt* OR disabilit* OR abnormal* OR restrict* OR problem* OR outcome* OR impair*)) OR (failure* ADJ3 thrive*) OR underdevelop* OR growth OR development* OR ((short* OR small) ADJ3 stature*) OR stunting OR (body ADJ3 (height* OR length* OR stature OR small OR weight OR mass* OR fat OR composition)) OR obes* OR overweight* OR adipos* OR bmi OR ((psychomot* OR motor* OR language* OR speech* OR psychosocial OR psycho-social OR psychologic*) ADJ3 (develop* OR delay*)) OR ((development* OR growth) ADJ3 delay*) OR neurodevelopment* OR ((child* OR infan*) ADJ3 development*) OR (grow* ADJ3 curve*) OR ((head OR cranial) ADJ3 circumference)).ab,ti.) AND (Screening/ OR exp risk factors/ OR risk assessment/ OR prediction/ OR prognosis/ OR ((risk ADJ3 (factor* OR high* OR assess*)) OR screen* OR predict* OR scoring OR rating OR scale*).ab,ti.) AND ((baby OR babies OR newborn* OR neonat* OR (birth* ADJ3 cohort*) OR postnatal* OR puerper* OR perinatal*).ab,ti.) AND (140.ag. OR (infan* OR (("2" OR two OR "1" OR one OR first OR second) ADJ2 year*)).ab,ti.) AND (exp Cohort Analysis/ OR exp Clinical Trials/ OR Followup Studies/ OR (cohort* OR longitudinal* OR retrospectiv* OR prospectiv* OR control* OR trial* OR follow up).ab,ti.) AND english.la. NOT (letter OR news OR comment OR editorial OR congresses OR abstracts).pt. NOT (deaf/ OR exp Hearing Disorders/ OR exp developing countries/ OR (deaf* OR hearing* OR Africa OR Asia OR ((developing OR underdevelop*) ADJ3 countr*)).ab,ti.) NOT (exp animals/ NOT humans/)

**Cochrane 547**

((((behav*) NEAR/3 (disorder* OR disturba* OR anomal* OR arrest* OR failure* OR retard* OR deficien* OR stunt* OR disabilit* OR abnormal* OR restrict* OR problem* OR outcome* OR impair*)) OR (failure* NEAR/3 thrive*) OR underdevelop* OR growth OR development* OR ((short* OR small) NEAR/3 stature*) OR stunting OR (body NEAR/3 (height* OR length* OR stature OR small OR weight OR mass* OR fat OR composition)) OR obes* OR overweight* OR adipos* OR bmi OR ((psychomot* OR motor* OR language* OR speech* OR psychosocial OR psycho-social OR psychologic*) NEAR/3 (develop* OR delay*)) OR ((development* OR growth) NEAR/3 delay*) OR neurodevelopment* OR ((child* OR infan*) NEAR/3 development*) OR (grow* NEAR/3 curve*) OR ((head OR cranial) NEAR/3 circumference)):ab,ti) AND (((risk NEAR/3 (factor* OR high* OR assess*)) OR screen* OR predict* OR scoring OR rating OR scale*):ab,ti) AND ((baby OR babies OR newborn* OR neonat* OR (birth* NEAR/3 cohort*) OR postnatal* OR puerper* OR perinatal*):ab,ti) AND ((infan* OR ((2 OR two OR 1 OR one OR first OR second) NEXT/2 year*)):ab,ti) AND ((cohort* OR longitudinal* OR retrospectiv* OR prospectiv* OR control* OR trial* OR 'follow up'):ab,ti)
