# Supplementary material for: Reducing growth and developmental problems in children: Development of an innovative postnatal risk assessment
Source: PLoS One. 2019 Jun 5;14(6):e0217261. doi: 10.1371/journal.pone.0217261 (PMC6550373; doi:10.1371/journal.pone.0217261)
Supplement: S2 File — (DOCX) [file pone.0217261.s002.docx]

**S2 File. Tables.**

Table A. Selected articles from the original literature search.

|  | **Author** | **Year of publication** | **Risk factor** | **Associated with** |
| --- | --- | --- | --- | --- |
| 1 | Alvik (1) | 2014 | Maternal depression, smoking | Development |
| 2 | Arpi (2) | 2014 | Prematurity | Development |
| 3 | Baptiste-Roberts (3) | 2011 | Gestational diabetes | Growth |
| 4 | Beaino (4) | 2011 | Prematurity | Development |
| 5 | Birbilis (5) | 2013 | Maternal obesity, smoking | Growth |
| 6 | Blustein (6) | 2013 | Maternal overweight | Growth |
| 7 | Cents (7) | 2013 | Maternal depression | Development |
| 8 | Conroy (8) | 2012 | Personality disorder, postnatal depression | Development |
| 9 | De Hoog (9) | 2011 | Gestational diabetes, ethnicity, (low) SES, LGA, maternal overweight, smoking | Growth |
| 10 | Deave (10) | 2008 | Maternal depression | Development |
| 11 | Durmus (11) | 2011 | Formula feeding | Catch-up growth |
| 12 | El Marroun (12) | 2011 | Cannabis, prematurity, smoking. | Development |
| 13 | El Marroun (13) | 2014 | Maternal depression | Development |
| 14 | Figueras (14) | 2009 | SGA | Development |
| 15 | Flores (15) | 2013 | Gestational diabetes, maternal overweight | Growth |
| 16 | Gaillard (16) | 2013 | Excessive maternal weight gain during pregnancy, maternal overweight, maternal underweight | Growth |
| 17 | Gao (17) | 2007 | Maternal psychological disorder, smoking | Development |
| 18 | Gibbs (18) | 2014 | Formula feeding | Growth |
| 19 | Gillman (19) | 2008 | Maternal overweight | Growth |
| 20 | Gutteling (20) | 2005 | Smoking | Development |
| 21 | Helderman (21) | 2012 | Prematurity, ethnicity, maternal overweight | Development |
| 22 | Henrichs (22) | 2012 | Hypothyroid | Development |
| 23 | Heppe (23) | 2013 | Maternal overweight, paternal overweight | Growth |
| 24 | Hinkle (24) | 2012 | Maternal overweight, maternal underweight | Growth |
| 25 | Hummel (25) | 2009 | Diabetes, LGA | Growth |
| 26 | Ino (26) | 2010 | Smoking | Growth |
| 27 | Jedrychowski (27) | 2011 | Maternal excessive weight gain in pregnancy, maternal overweight | Growth |
| 28 | Jordan (28) | 2005 | SGA | Catch-up growth |
| 29 | Kakinami (29) | 2014 | Low SES | Growth |
| 30 | Karaolis-Danckert (30) | 2008 | Formula feeding | Growth |
| 31 | Kerstjens (31) | 2012 (August) | Prematurity, SGA | Development |
| 32 | Kerstjens (32) | 2013 | Prematurity, maternal overweight | Development |
| 33 | Kerstjens (33) | 2012 (December) | Prematurity | Development |
| 34 | Kiechl-Kohlendorfer (34) | 2010 | Prematurity, smoking | Development |
| 35 | Knudsen (35) | 2014 | Alcohol | Development |
| 36 | Koutra (36) | 2013 | Maternal depression, postnatal depression | Development |
| 37 | Lewis (37) | 2011 | Cocaine | Development |
| 38 | Li (38) | 2007 | LGA, maternal overweight | Growth |
| 39 | Moller (39) | 2014 | Excessive maternal weight gain during pregnancy, maternal overweight, smoking |  |
| 40 | Morinis (40) | 2013 | Teen mom | Development |
| 41 | Noten (41) | 2015 | Hypothyroid | Development |
| 42 | Odd (42) | 2013 | Prematurity | Development |
| 43 | Oddy (43) | 2010 | Formula feeding, Depression, smoking | Development |
| 44 | Oken (44) | 2005 | Smoking | Growth |
| 45 | Pan (45) | 2013 | Low SES, ethnicity | Growth |
| 46 | Peralta-Carcelen (46) | 2013 | SGA, low SES | Development |
| 47 | Pham (47) | 2013 | LGA, maternal overweight | Growth |
| 48 | Power (48) | 2010 | Smoking | Growth |
| 49 | Reeske (49) | 2013 | Ethnicity, smoking | Catch-up growth |
| 50 | Rijlaarsdam (50) | 2013 | Ethnicity, low SES | Development |
| 51 | Robinson (51) | 2013 | Prematurity | Development |
| 52 | Roza (52) | 2009 | Ethnicity, low SES, smoking | Development |
| 53 | Salsberry (53) | 2005 | Maternal overweight, smoking, prematurity | Growth |
| 54 | Samra (54) | 2011 | Prematurity | Development |
| 55 | Schjolberg (55) | 2011 | SGA, ethnicity, low Apgar score, low SES, prematurity, non-native speaking parents | Development |
| 56 | Skurtveit (56) | 2014 | Maternal depression, SSRI medication | Development |
| 57 | Talge (57) | 2010 | Prematurity | Development |
| 58 | Timmermans (58) | 2014 | Smoking | Growth, catch-up growth |
| 59 | Tong (59) | 2006 | SGA | Development |
| 60 | Twells (60) | 2010 | Formula feeding | Growth |
| 61 | Van Rossem (61) | 2014 | Ethnicity | Growth |
| 62 | Veiby (62) | 2013 | Antiepileptic medication | Development |
| 63 | Weden (63) | 2012 | Low SES | Growth |
| 64 | Wen (64) | 2014 | Maternal overweight | Growth |
| 65 | Weng (65) | 2012 | Formula feeding, smoking | Growth |
| 66 | Weyerman (66) | 2006 | Formula feeding | Growth |
| 67 | Xiong (67) | 2006 | SGA, LGA | Catch-up growth |
| 68 | Zhu (68) | 2012 | Prematurity | Development |
| 69 | Zwicker (69) | 2013 | SGA | Development |

Table B. Selected articles from the additional literature search.

|  | **Author** | **Year of publication** | **Risk factor** | **Associated with** |
| --- | --- | --- | --- | --- |
|  | Bilic-Kirin (70) | 2014 | Socioeconomic status, maternal unemployment | Growth |
|  | Bradley (71) | 2002 | Socioeconomic status, single parent, family conflict | Development |
|  | Crockenberg (72) | 1981 | Lack of social support | Development |
|  | Gilbert (73) | 2013 | Home violence | Development |
|  | Stewart (74) | 2012 | Maternal unemployment | Growth |
|  | Yeung (75) | 2002 | Financial problems | Development |
|  | Gershoff (76) | 2007 | Financial problems, unsafe home environment, no insurance | Development |
|  | Masten (77) | 1993 | Being homeless/unsafe home environment | Development |
|  | Van der Heide (78) | 2010 | Single parent | Development |

Table C. Quotes from focus group participants on the development of the postnatal R4U.

| 1. Implementation of the assessment:    1. A consultation has an added value over questionnaires because of its possibility to explain the content.    2. Privacy should be taken into account.    3. There seems to be a trend of parents who are reluctant to share their data with PCHC, possibly because they don’t want their vulnarabilty to become public.    4. Not all parents know which diagnostic tests are done to their child in the hospital.    5. PCHC organizations have different definitions of prematurity and the care for premature born children is differently organized.    6. A postnatal home consultation takes place with every child, as well after discharge from the hospital    7. There are important risk factors which are not part of the basic dataset. These could be analyzed with the postnatal R4U.    8. I suggest to take the experience of the parent into account as well.    9. I see a possibility for the postnatal home visit to ask for informed consent and ask some additional questions.    10. I would assess which regular consultations are similar in different PCHC locations/municipalities. This can vary widely.    11. I would take already developed protocols and guidelines into account.    12. Parents become increasingly critical when it comes to saving data at governmental institutions. Some parents object to creating a digital patient file at the PCHC.    13. Training in motivational speaking could aid nurses to be more confident in counseling for the research project.    14. Parents sometimes don’t understand the patient information leaflet.    15. An inclusion fee could be risky. You can put a lot of effort in including patients, but still don’t reach the predefined limit.    16. I would suggest to ask parents what they need, this is included in other questionnaires as well.    17. There is a lot of ambiguity when it comes to parents with a low IQ. How do you assess it and what can you do about it?    18. The same accounts for illiteracy. |
| --- |
| 1. Growth:    1. Disharmonic growth is the most dangerous kind, especially in the first three months of life.    2. Catch-up growth is a difficult definition and not easy to measure. The literature is not ambiguous.    3. The measurement of height with young children is subject to bias, due to intra-observer and inter-observer variation.    4. I suggest to add the head circumference in the research project. |
| 1. Development:    1. There is a big difference in language development disorder and lack of stimulation.    2. I suggest to consider questionnaires such as the ASQ or CBCL in the research project, to measure development.    3. Language or speech developmental delay can in reality be detected starting with the age of 2 years old. |
| 1. Social risk factors:    1. Considering maternal high workload, you could look at mothers who work in shifts.    2. In research little associations are found considering high workload and being a single parent.    3. On the contrary, more associations are found in height of the father, parity and the age of the mother.    4. Family income and paternal income are very defining for the health of the child.    5. Level of education should be considered as a risk factor.    6. Work status and unemployment are very important risk factors and are related with low socio economic status.    7. You could design a scale model for socioeconomic status and combine multiple factors.    8. Low income, below 1000 euros a month should be considered in the assessment as well.    9. Teen pregnancies are a huge risk.    10. Age of the mother should be considered above the age of 40.    11. There is a big ethnic diversity in the Netherlands.    12. Considering ethnicity you should well define what you are going to measure.    13. Humidity and fungus are probably more related to low socio economic status.    14. If possible, you could consider air pollution as a risk factor.    15. Child marriages and arranged marriages impose a risk.    16. I would consult the foundation on reading and writing when it comes to risk factors concerning speech and language development.    17. Stress doesn’t have to be the same thing as experiencing a high workload.    18. Stress is a difficult definition to grasp.    19. In high SES there is more alcohol abuse.    20. High SES comes with high pressure to perform well and parents often don’t adjust their lifestyle when children come into the family.    21. My experience in practice is that high SES children are frequently restless.    22. Excessive crying in babies is more frequently seen in high SES families.    23. Low SES children respond more calmly.    24. Low SES families have a larger network in their close vicinity.    25. Postal code is not the most reliable item to assess SES with.    26. There is a big difference in first and third generation immigrants.    27. Having a large social network is an important protecting factor.    28. The quality of the social network is also important. Two people could be of better support than ten other people.    29. You could ask the parent what kind of childhood they had themselves, did they get the right example of their parents? There is a lot of evidence about the wrong example from parents and risks for the child.    30. Multilingualism could be a protecting factor as well as a risk factor. For gifted children it could be an advantage, for others it could decrease development.    31. Children who speak their parents native language well but don’t speak Dutch very well (yet), do not have impaired development. |
| 1. Lifestyle:    1. In some research they find a positive association with alcohol, because of the confounding effect with a high socio economic status.    2. The problem with alcohol is that there is no safe detectable bottom-limit.    3. People who smoke incidentally, usually don’t smoke during pregnancy.    4. Drug use during pregnancy obviously has a negative effect on the child.    5. It seems better not to separate the use of soft drugs and hard drugs, since cannabis abuse shows evident effects.    6. For the development of a risk score is seems better to take all drugs together.    7. Gambling addiction is a risk factor as well.    8. With a large amount of animals in the household, there is bad hygiene and less finances for basic life support.    9. In PCHC, BMI of the mother is not asked during a consultation, only the height of the mother is discussed.    10. Weight is a painful question, it should be available from the handover from the midwife.    11. Lifestyle includes: are you able to implement structure and regularity into your daily rhythm? That’s an important factor on how things are organized at home. This could influence your adult life.    12. The previous mentioned could be difficult to assess within 6 weeks postnatally. Parents are often searching for structure in their new life until then.    13. The amount of people in an household could be a risk factor. For example, three families in one house. A too small living environment. This could show during a home visit by the PCHC nurse.    14. Could music be a protective factor? |
| 1. Medical:    1. Opiates are considered to have a negative effect on child’s health.    2. Most research on the use of antidepressants in pregnancy show (almost) no association with a child’s development.    3. An embryo is dependent on the thyroid hormone of the mother. The pituitary gland starts being active around 20 weeks of gestation.    4. The influence of depression and psychiatric diseases on the child seems to be transgenerational and possibly epigenetic. Not only is there a relationship between mother and child, but also a relationship between father and child and a relationship between previous generation and the child.    5. The influence of depression could also have a causally related. It could have added to insufficient parenting techniques in several generations.    6. Ace inhibitors are known to influence the development of the kidneys, not the development of the brain.    7. I would suggest to combine all eating disorders, for the risk assessment.    8. There is an increasing prevalence of women who underwent bariatric surgery before pregnancy. Their children have an increased risk of malnutrition as well as overfeeding. For now, this group is small.    9. Children from mothers with diabetes gravidarum are born with a bonus for height and weight, and take it with them in the upcoming years. They remain to grow parallel on the top of the growth curve.    10. Deaf or hearing impaired parents, seems to influence speech and language development of the child.    11. Severely ill father or mother and the other parent becomes caregiver, hence the child receives less attention or could become a primary caregiver as well.    12. Dyslexia in parents could influence speech and language development of the child. |
| 1. Obstetrical:    1. I suggest to choose between formula feeding as a risk factor or breastfeeding as a protecting factor, not both.    2. First born children are often born a bit smaller, so they show more catch-up growth.    3. The second, third etc children are born a bit more heavier.    4. Catch-up growth depends on the gestational age at birth. If you’re born closer to 40 weeks of gestation, the smaller the chance for catch-up growth.    5. Reproductive assisted technology seems to be associated with developmental problems, there should be evidence in scientific literature.    6. I suggest to consider the Institute of Medicine criteria for gestational weight gain on the weight gain in pregnancy. This depends on the weight before pregnancy.    7. I suggest to make clear definitions for small for gestational age, prematurity and low Apgar score.    8. I suggest make categories of prematurity: extreme premature under 27 weeks of gestation, premature in two categories from 27 weeks until 32 weeks and 32 weeks until 34 weeks, late premature from 34 weeks until 37 weeks of gestation.    9. Late premature born children and children who are born at 37 until 38 weeks of gestation are an interesting group which could well identified in PCHC.    10. Asphyxia is an important factor for later outcome in post term birth (above 42 weeks of gestation).    11. Hypoglycemia in premature born children is an important factor in later outcome.    12. De health outcomes for children born with congenital anomalies seems to be associated with the severity of the anomaly and the necessary medical care.    13. Caesarean sections seems to be associated with the risk of childhood obesity.    14. Breech position might be associated with low thyroid function of the mother. The baby does not think of turning upside down.    15. Formula feeding might be only a risk factor in combination with other risk factors for obesity?    16. Other risk factors could be pre-eclampsia and HELLP syndrome.    17. Could a traumatic delivery be a risk factor?    18. Mothers of premature born children are often better motivated to breastfeed.    19. Mother-child bonding is difficult to measure in PCHC, especially until six weeks postnatally.    20. Is there research done to compare term born LGA children with premature born LGA children?    21. An infection during delivery and after birth can influence the development of the child. Especially in children who had meningitis.    22. Children who were born after prematurely ruptured membranes re admitted longer at the NICU, which increases the chance of developmental problems.    23. I would suggest to balance protective factors against risk factors. In PCHC practice there is more focus on positive factors.    24. In the PCHC digital file there is an item called: “what kind of child is this?” |

Table D. Quotes from focus group participants on the development of the care pathways.

| 1. Care pathways:    1. The most important thing is to have a good overview of the community (care) resources.    2. Social work could assist in creating the overview of the community (care) resources.    3. When developing care pathways I would suggest to also use national and organizational guidelines regarding that subject.    4. In a care pathway it is important to add the appointment of a case manager.    5. PCHC seems more appropriate as case manager because maternity care has a short care agreement with the family.    6. Maternity care could provide important information because they’re primary working in the household.    7. There seem to be a lot organizations who work separately and are not informed about the others existence. |
| --- |
| 1. Psychosocial:    1. A care pathway for illegal persons seems of more value than a care pathway on people who don’t have insurance.    2. Often there are more problems than one, when someone has financial issues they often have housing problems, for example.    3. A lot of different social care pathways seems redundant, because you would like to connect them all to each other.    4. It’s starting to become common practice to have multidisciplinary consultations together with the parents of the child. |
| 1. Growth problems:    1. I would suggest to start a care pathway in case of BMI of the mother is above 30, not above 25. Otherwise you would create a very large target population for the care pathway. This is not feasible.    2. In this care pathway I would also add children who are born small for gestational age (SGA). These children often develop long-term complications because of overweight.    3. In this care pathway parental factors are important as well.    4. I would suggest to also focus on children who show catch-up growth after birth. |
| 1. Smoking/alcohol/drugs:    1. Smoking in the environment of the child is also an important risk factors, but maybe a bit more difficult to find out.    2. The use of drugs by one or both parents should justify a report to child welfare services.    3. In case of drug abuse and/or contact with child welfare services you could add in the care pathway: ‘action taken during pregnancy?’. In case of no action or care during pregnancy, action postnatally could be indicated.    4. In case of substance abuse, there is an indication for an addiction program.    5. In case of parental smoking, the risk of obesity of the child should be taken into account. |
| 1. (Chronical) illness of a parent:    1. Hypothyroid disease of the mother during pregnancy is difficult to find out for PCHC professionals.    2. Chronical illness of a parent could result in less attention to the child. It doesn’t matter which disease, the burden for the family is the most important aspect.    3. In case of chronical illness the family composition and care for the children are the most important aspects.    4. It is important to have a good overview of what care arrangements are already taken. |
| 1. Psychiatry:    1. For psychiatric disease of the parent I would suggest to design a separate care pathway.    2. I would suggest to use the Edinburgh Postnatal Depression Scale in this care pathway. And possibly combine this with ‘excessive crying’.    3. An excessive crying baby is an indication to watch out for depression in the mother. |
| 1. Congenital anomalies:    1. I would definitely add congenital anomalies to the intervention concerning the impact on parents and the family.    2. One visible anomaly is an indication to perform additional research for more anomalies.    3. Congenital anomalies could predict future developmental problems.    4. You could add the postnatal screening protocol to the intervention. |
| 1. Prematurity and small for gestational age:    1. I would suggest to involve regional aftercare facilities (in which PCHC collaborates with pediatric care) during the development of the care pathway. |
| 1. Developmental problems:    1. In care pathways one could direct to developmental protocols, guidelines or questionnaires. |

1. Alvik A. Variables predicting low infant developmental scores: maternal age above 30 years is a main predictor. Scand J Public Health 2014;42(2):113-9.

2. Arpi E, Ferrari F. Preterm birth and behaviour problems in infants and preschool-age children: a review of the recent literature. Dev Med Child Neurol 2013;55(9):788-96.

3. Baptiste-Roberts K, Nicholson WK, Wang NY, Brancati FL. Gestational diabetes and subsequent growth patterns of offspring: the National Collaborative Perinatal Project. Matern Child Health J 2012;16(1):125-32.

4. Beaino G, Khoshnood B, Kaminski M, Marret S, Pierrat V, Vieux R, et al. Predictors of the risk of cognitive deficiency in very preterm infants: the EPIPAGE prospective cohort. Acta Paediatr 2011;100(3):370-8.

5. Birbilis M, Moschonis G, Mougios V, Manios Y, Healthy Growth Study g. Obesity in adolescence is associated with perinatal risk factors, parental BMI and sociodemographic characteristics. Eur J Clin Nutr 2013;67(1):115-21.

6. Blustein J, Attina T, Liu M, Ryan AM, Cox LM, Blaser MJ, et al. Association of caesarean delivery with child adiposity from age 6 weeks to 15 years. Int J Obes (Lond) 2013;37(7):900-6.

7. Cents RA, Diamantopoulou S, Hudziak JJ, Jaddoe VW, Hofman A, Verhulst FC, et al. Trajectories of maternal depressive symptoms predict child problem behaviour: the Generation R study. Psychol Med 2013;43(1):13-25.

8. Conroy S, Pariante CM, Marks MN, Davies HA, Farrelly S, Schacht R, et al. Maternal psychopathology and infant development at 18 months: the impact of maternal personality disorder and depression. J Am Acad Child Adolesc Psychiatry 2012;51(1):51-61.

9. de Hoog ML, van Eijsden M, Stronks K, Gemke RJ, Vrijkotte TG. Overweight at age two years in a multi-ethnic cohort (ABCD study): the role of prenatal factors, birth outcomes and postnatal factors. BMC Public Health 2011;11:611.

10. Deave T, Heron J, Evans J, Emond A. The impact of maternal depression in pregnancy on early child development. Bjog 2008;115(8):1043-51.

11. Durmus B, van Rossem L, Duijts L, Arends LR, Raat H, Moll HA, et al. Breast-feeding and growth in children until the age of 3 years: the Generation R Study. Br J Nutr 2011;105(11):1704-11.

12. El Marroun H, Hudziak JJ, Tiemeier H, Creemers H, Steegers EA, Jaddoe VW, et al. Intrauterine cannabis exposure leads to more aggressive behavior and attention problems in 18-month-old girls. Drug Alcohol Depend 2011;118(2-3):470-4.

13. El Marroun H, White TJ, van der Knaap NJ, Homberg JR, Fernandez G, Schoemaker NK, et al. Prenatal exposure to selective serotonin reuptake inhibitors and social responsiveness symptoms of autism: population-based study of young children. Br J Psychiatry 2014;205(2):95-102.

14. Figueras F, Cruz-Martinez R, Sanz-Cortes M, Arranz A, Illa M, Botet F, et al. Neurobehavioral outcomes in preterm, growth-restricted infants with and without prenatal advanced signs of brain-sparing. Ultrasound Obstet Gynecol 2011;38(3):288-94.

15. Flores G, Lin H. Factors predicting severe childhood obesity in kindergarteners. Int J Obes (Lond) 2013;37(1):31-9.

16. Gaillard R, Durmus B, Hofman A, Mackenbach JP, Steegers EA, Jaddoe VW. Risk factors and outcomes of maternal obesity and excessive weight gain during pregnancy. Obesity (Silver Spring) 2013;21(5):1046-55.

17. Gao W, Paterson J, Abbott M, Carter S, Iusitini L. Maternal mental health and child behaviour problems at 2 years: findings from the Pacific Islands Families Study. Aust N Z J Psychiatry 2007;41(11):885-95.

18. Gibbs BG, Forste R. Socioeconomic status, infant feeding practices and early childhood obesity. Pediatr Obes 2014;9(2):135-46.

19. Gillman MW. Prenatal famine and developmental origins of type 2 diabetes. Lancet Diabetes Endocrinol 2015;3(10):751-2.

20. Gutteling BM, de Weerth C, Willemsen-Swinkels SH, Huizink AC, Mulder EJ, Visser GH, et al. The effects of prenatal stress on temperament and problem behavior of 27-month-old toddlers. Eur Child Adolesc Psychiatry 2005;14(1):41-51.

21. Helderman JB, O'Shea TM, Kuban KC, Allred EN, Hecht JL, Dammann O, et al. Antenatal antecedents of cognitive impairment at 24 months in extremely low gestational age newborns. Pediatrics 2012;129(3):494-502.

22. Henrichs J, Ghassabian A, Peeters RP, Tiemeier H. Maternal hypothyroxinemia and effects on cognitive functioning in childhood: how and why? Clin Endocrinol (Oxf) 2013;79(2):152-62.

23. Heppe DH, Kiefte-de Jong JC, Durmus B, Moll HA, Raat H, Hofman A, et al. Parental, fetal, and infant risk factors for preschool overweight: the Generation R Study. Pediatr Res 2013;73(1):120-7.

24. Hinkle SN, Schieve LA, Stein AD, Swan DW, Ramakrishnan U, Sharma AJ. Associations between maternal prepregnancy body mass index and child neurodevelopment at 2 years of age. Int J Obes (Lond) 2012;36(10):1312-9.

25. Hummel S, Pfluger M, Kreichauf S, Hummel M, Ziegler AG. Predictors of overweight during childhood in offspring of parents with type 1 diabetes. Diabetes Care 2009;32(5):921-5.

26. Ino T. Maternal smoking during pregnancy and offspring obesity: meta-analysis. Pediatr Int 2010;52(1):94-9.

27. Jedrychowski W, Maugeri U, Kaim I, Budzyn-Mrozek D, Flak E, Mroz E, et al. Impact of excessive gestational weight gain in non-smoking mothers on body fatness in infancy and early childhood. Prospective prebirth cohort study in Cracow. J Physiol Pharmacol 2011;62(1):55-64.

28. Jordan IM, Robert A, Francart J, Sann L, Putet G. Growth in extremely low birth weight infants up to three years. Biol Neonate 2005;88(1):57-65.

29. Kakinami L, Seguin L, Lambert M, Gauvin L, Nikiema B, Paradis G. Poverty's latent effect on adiposity during childhood: evidence from a Quebec birth cohort. J Epidemiol Community Health 2014;68(3):239-45.

30. Karaolis-Danckert N, Buyken AE, Kulig M, Kroke A, Forster J, Kamin W, et al. How pre- and postnatal risk factors modify the effect of rapid weight gain in infancy and early childhood on subsequent fat mass development: results from the Multicenter Allergy Study 90. Am J Clin Nutr 2008;87(5):1356-64.

31. Kerstjens JM, Bocca-Tjeertes IF, de Winter AF, Reijneveld SA, Bos AF. Neonatal morbidities and developmental delay in moderately preterm-born children. Pediatrics 2012;130(2):e265-72.

32. Kerstjens JM, de Winter AF, Sollie KM, Bocca-Tjeertes IF, Potijk MR, Reijneveld SA, et al. Maternal and pregnancy-related factors associated with developmental delay in moderately preterm-born children. Obstet Gynecol 2013;121(4):727-33.

33. Kerstjens JM, de Winter AF, Bocca-Tjeertes IF, Bos AF, Reijneveld SA. Risk of developmental delay increases exponentially as gestational age of preterm infants decreases: a cohort study at age 4 years. Dev Med Child Neurol 2012;54(12):1096-101.

34. Kiechl-Kohlendorfer U, Ralser E, Pupp Peglow U, Reiter G, Griesmaier E, Trawoger R. Smoking in pregnancy: a risk factor for adverse neurodevelopmental outcome in preterm infants? Acta Paediatr 2010;99(7):1016-9.

35. Knudsen AK, Skogen JC, Ystrom E, Sivertsen B, Tell GS, Torgersen L. Maternal pre-pregnancy risk drinking and toddler behavior problems: the Norwegian Mother and Child Cohort Study. Eur Child Adolesc Psychiatry 2014;23(10):901-11.

36. Koutra K, Chatzi L, Bagkeris M, Vassilaki M, Bitsios P, Kogevinas M. Antenatal and postnatal maternal mental health as determinants of infant neurodevelopment at 18 months of age in a mother-child cohort (Rhea Study) in Crete, Greece. Soc Psychiatry Psychiatr Epidemiol 2013;48(8):1335-45.

37. Lewis BA, Minnes S, Short EJ, Weishampel P, Satayathum S, Min MO, et al. The effects of prenatal cocaine on language development at 10 years of age. Neurotoxicol Teratol 2011;33(1):17-24.

38. Li C, Goran MI, Kaur H, Nollen N, Ahluwalia JS. Developmental trajectories of overweight during childhood: role of early life factors. Obesity (Silver Spring) 2007;15(3):760-71.

39. Moller SE, Ajslev TA, Andersen CS, Dalgard C, Sorensen TI. Risk of childhood overweight after exposure to tobacco smoking in prenatal and early postnatal life. PLoS One 2014;9(10):e109184.

40. Morinis J, Carson C, Quigley MA. Effect of teenage motherhood on cognitive outcomes in children: a population-based cohort study. Arch Dis Child 2013;98(12):959-64.

41. Noten AM, Loomans EM, Vrijkotte TG, van de Ven PM, van Trotsenburg AS, Rotteveel J, et al. Maternal hypothyroxinaemia in early pregnancy and school performance in 5-year-old offspring. Eur J Endocrinol 2015;173(5):563-71.

42. Odd DE, Lingam R, Emond A, Whitelaw A. Movement outcomes of infants born moderate and late preterm. Acta Paediatr 2013;102(9):876-82.

43. Oddy WH, Kendall GE, Li J, Jacoby P, Robinson M, de Klerk NH, et al. The long-term effects of breastfeeding on child and adolescent mental health: a pregnancy cohort study followed for 14 years. J Pediatr 2010;156(4):568-74.

44. Oken E, Huh SY, Taveras EM, Rich-Edwards JW, Gillman MW. Associations of maternal prenatal smoking with child adiposity and blood pressure. Obes Res 2005;13(11):2021-8.

45. Pan L, May AL, Wethington H, Dalenius K, Grummer-Strawn LM. Incidence of obesity among young U.S. children living in low-income families, 2008-2011. Pediatrics 2013;132(6):1006-13.

46. Peralta-Carcelen M, Bailey K, Rector R, Gantz M, Network NNR. Behavioral and socioemotional competence problems of extremely low birth weight children. J Perinatol 2013;33(11):887-92.

47. Pham MT, Brubaker K, Pruett K, Caughey AB. Risk of childhood obesity in the toddler offspring of mothers with gestational diabetes. Obstet Gynecol 2013;121(5):976-82.

48. Power C, Atherton K, Thomas C. Maternal smoking in pregnancy, adult adiposity and other risk factors for cardiovascular disease. Atherosclerosis 2010;211(2):643-8.

49. Reeske A, Spallek J, Bammann K, Eiben G, De Henauw S, Kourides Y, et al. Migrant background and weight gain in early infancy: results from the German study sample of the IDEFICS study. PLoS One 2013;8(4):e60648.

50. Rijlaarsdam J, Tiemeier H, Hofman A, Jaddoe VW, Mackenbach JP, Verhulst FC, et al. Home environments of infants: relations with child development through age 3. J Epidemiol Community Health 2013;67(1):14-20.

51. Robinson M, Whitehouse AJ, Zubrick SR, Pennell CE, Jacoby P, McLean NJ, et al. Delivery at 37 weeks' gestation is associated with a higher risk for child behavioural problems. Aust N Z J Obstet Gynaecol 2013;53(2):143-51.

52. Roza SJ, Verhulst FC, Jaddoe VW, Steegers EA, Mackenbach JP, Hofman A, et al. Maternal smoking during pregnancy and child behaviour problems: the Generation R Study. Int J Epidemiol 2009;38(3):680-9.

53. Salsberry PJ, Reagan PB. Dynamics of early childhood overweight. Pediatrics 2005;116(6):1329-38.

54. Samra HA, McGrath JM, Wehbe M. An integrated review of developmental outcomes and late-preterm birth. J Obstet Gynecol Neonatal Nurs 2011;40(4):399-411.

55. Schjolberg S, Eadie P, Zachrisson HD, Oyen AS, Prior M. Predicting language development at age 18 months: data from the Norwegian Mother and Child Cohort Study. J Dev Behav Pediatr 2011;32(5):375-83.

56. Skurtveit S, Selmer R, Roth C, Hernandez-Diaz S, Handal M. Prenatal exposure to antidepressants and language competence at age three: results from a large population-based pregnancy cohort in Norway. Bjog 2014;121(13):1621-31.

57. Talge NM, Holzman C, Wang J, Lucia V, Gardiner J, Breslau N. Late-preterm birth and its association with cognitive and socioemotional outcomes at 6 years of age. Pediatrics 2010;126(6):1124-31.

58. Timmermans SH, Mommers M, Gubbels JS, Kremers SP, Stafleu A, Stehouwer CD, et al. Maternal smoking during pregnancy and childhood overweight and fat distribution: the KOALA Birth Cohort Study. Pediatr Obes 2014;9(1):e14-25.

59. Tong S, Baghurst P, McMichael A. Birthweight and cognitive development during childhood. J Paediatr Child Health 2006;42(3):98-103.

60. Twells L, Newhook LA. Can exclusive breastfeeding reduce the likelihood of childhood obesity in some regions of Canada? Can J Public Health 2010;101(1):36-9.

61. van Rossem L, Hafkamp-de Groen E, Jaddoe VW, Hofman A, Mackenbach JP, Raat H. The role of early life factors in the development of ethnic differences in growth and overweight in preschool children: a prospective birth cohort. BMC Public Health 2014;14:722.

62. Veiby G, Daltveit AK, Schjolberg S, Stoltenberg C, Oyen AS, Vollset SE, et al. Exposure to antiepileptic drugs in utero and child development: a prospective population-based study. Epilepsia 2013;54(8):1462-72.

63. Weden MM, Brownell P, Rendall MS. Prenatal, perinatal, early life, and sociodemographic factors underlying racial differences in the likelihood of high body mass index in early childhood. Am J Public Health 2012;102(11):2057-67.

64. Wen LM, Baur LA, Rissel C, Xu H, Simpson JM. Correlates of body mass index and overweight and obesity of children aged 2 years: findings from the healthy beginnings trial. Obesity (Silver Spring) 2014;22(7):1723-30.

65. Weng SF, Redsell SA, Swift JA, Yang M, Glazebrook CP. Systematic review and meta-analyses of risk factors for childhood overweight identifiable during infancy. Arch Dis Child 2012;97(12):1019-26.

66. Weyermann M, Rothenbacher D, Brenner H. Duration of breastfeeding and risk of overweight in childhood: a prospective birth cohort study from Germany. Int J Obes (Lond) 2006;30(8):1281-7.

67. Xiong X, Wightkin J, Magnus JH, Pridjian G, Acuna JM, Buekens P. Birth weight and infant growth: optimal infant weight gain versus optimal infant weight. Matern Child Health J 2007;11(1):57-63.

68. Zhu JL, Olsen J, Olesen AW. Risk for developmental coordination disorder correlates with gestational age at birth. Paediatr Perinat Epidemiol 2012;26(6):572-7.

69. Zwicker JG, Yoon SW, Mackay M, Petrie-Thomas J, Rogers M, Synnes AR. Perinatal and neonatal predictors of developmental coordination disorder in very low birthweight children. Arch Dis Child 2013;98(2):118-22.

70. Bilic-Kirin V, Gmajnic R, Burazin J, Milicic V, Buljan V, Ivanko M. Association between socioeconomic status and obesity in children. Coll Antropol 2014;38(2):553-8.

71. Bradley RH, Corwyn RF. Socioeconomic status and child development. Annu Rev Psychol 2002;53:371-99.

72. Crockenberg SB. Infant Irritability, Mother Responsiveness, and Social Support Influences on the Security of Infant-Mother Attachment. Child Development 1981;52(3, (september 1981)):857-865.

73. Gilbert AL, Bauer NS, Carroll AE, Downs SM. Child exposure to parental violence and psychological distress associated with delayed milestones. Pediatrics 2013;132(6):e1577-83.

74. Stewart L, Liu Y, Rodriguez E. Maternal unemployment and childhood overweight: is there a relationship? J Epidemiol Community Health 2012;66(7):641-6.

75. Yeung WJ, Linver MR, Brooks-Gunn J. How money matters for young children's development: parental investment and family processes. Child Dev 2002;73(6):1861-79.

76. Gershoff ET, Aber JL, Raver CC, Lennon MC. Income is not enough: incorporating material hardship into models of income associations with parenting and child development. Child Dev 2007;78(1):70-95.

77. Masten AS, Miliotis D, Graham-Bermann SA, Ramirez M, Neemann J. Children in homeless families: risks to mental health and development. J Consult Clin Psychol 1993;61(2):335-43.

78. van der Heide K. Thuiswonende deelnemers uit éénoudergezin presteren minder in het middelbaar beroepsonderwijs. Sociaaleconomische trends, 1e kwartaal 2010 2010.
