# Supplementary figures and images for: Reducing growth and developmental problems in children: Development of an innovative postnatal risk assessment
Source: PLoS One. 2019 Jun 5;14(6):e0217261. doi: 10.1371/journal.pone.0217261 (PMC6550373; doi:10.1371/journal.pone.0217261)

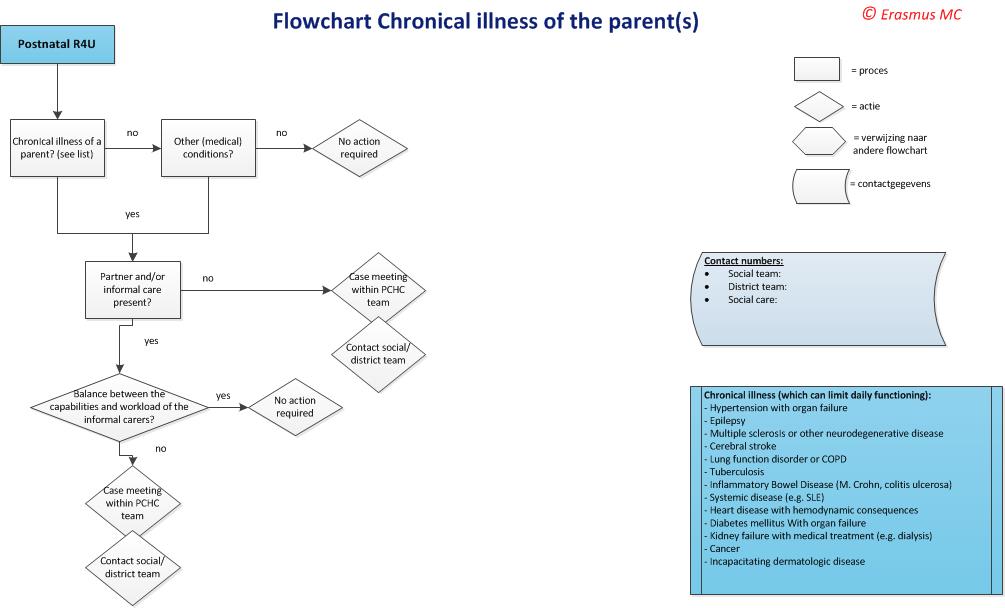

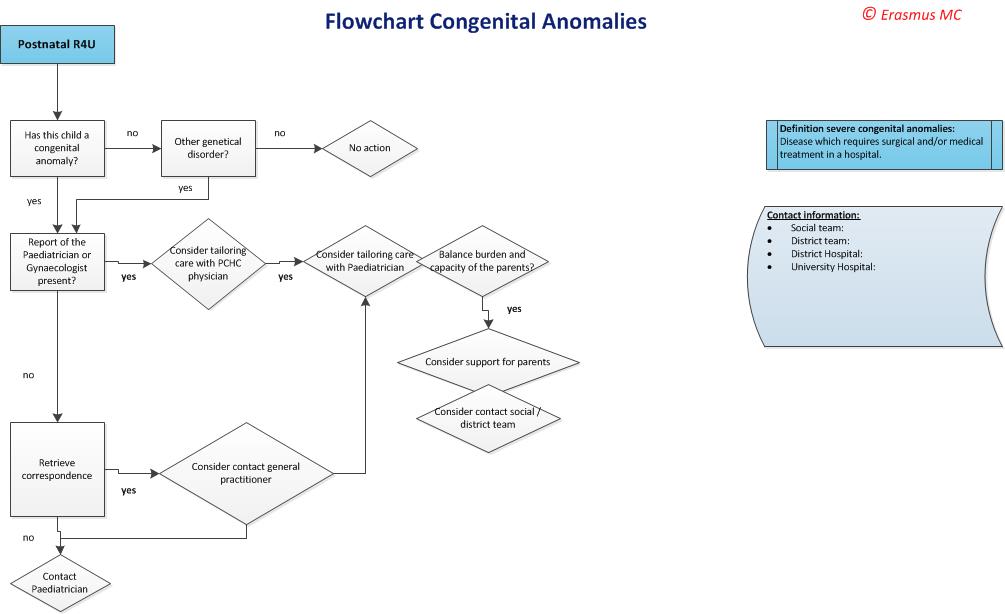

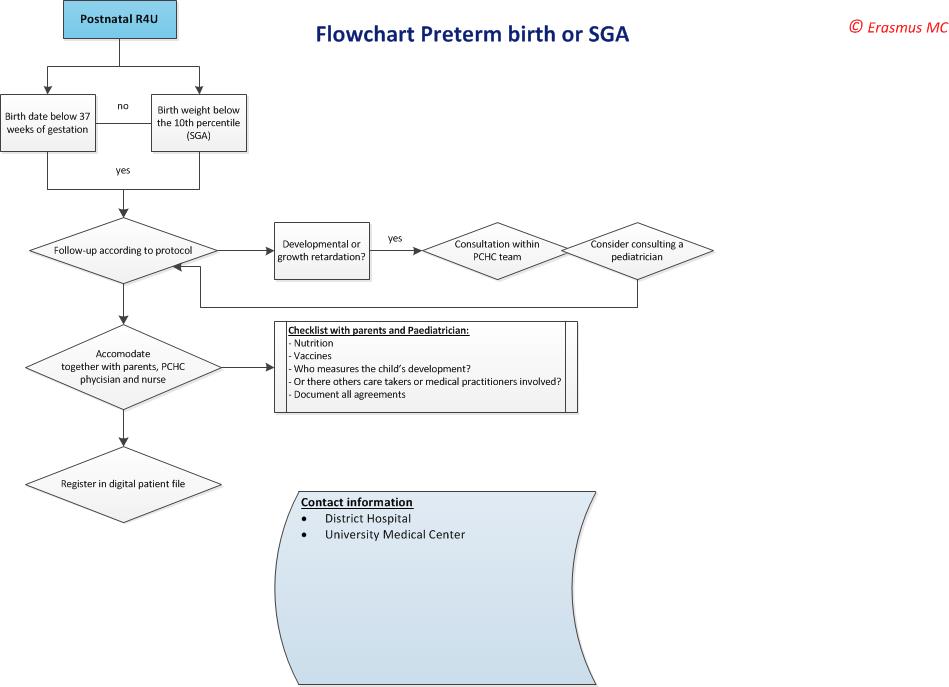

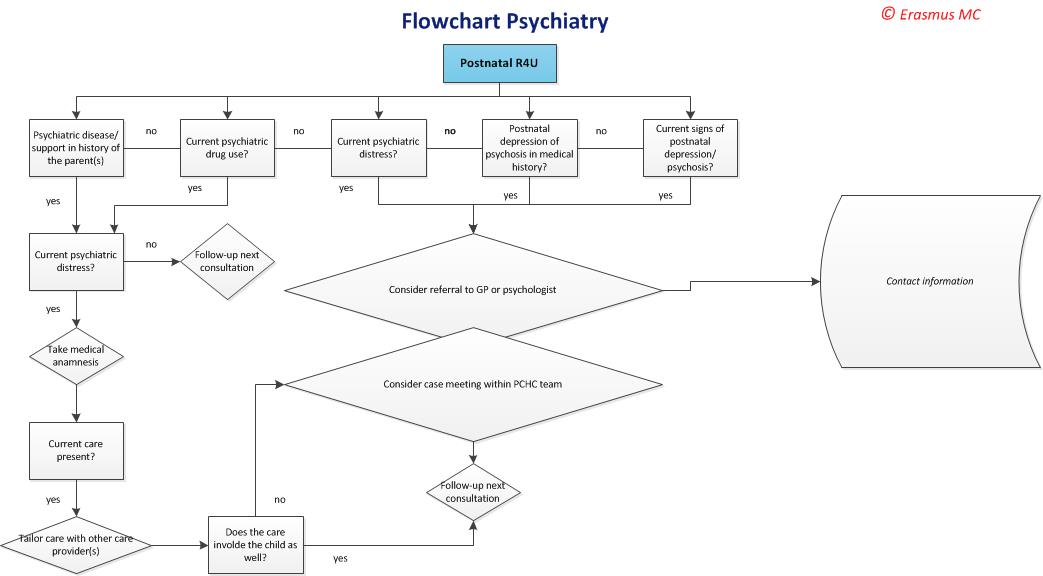

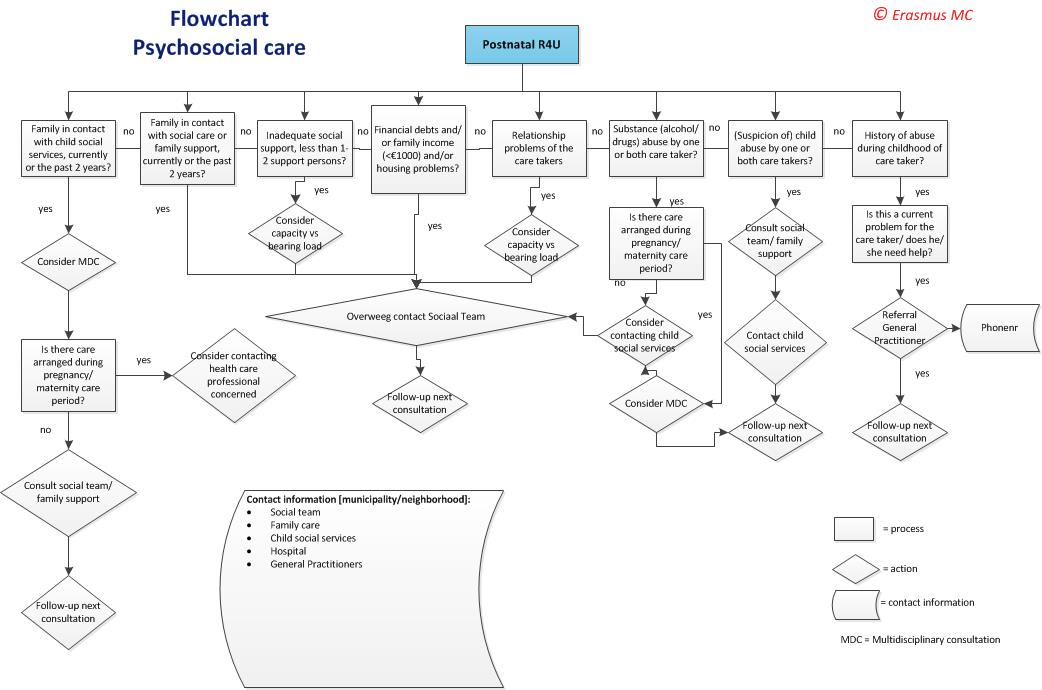

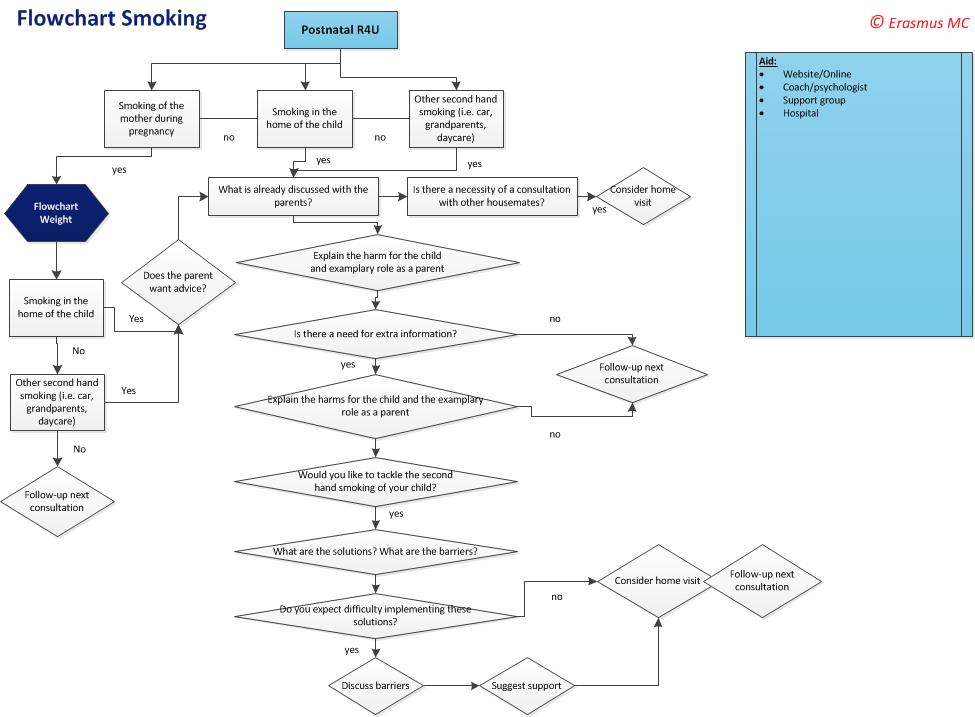

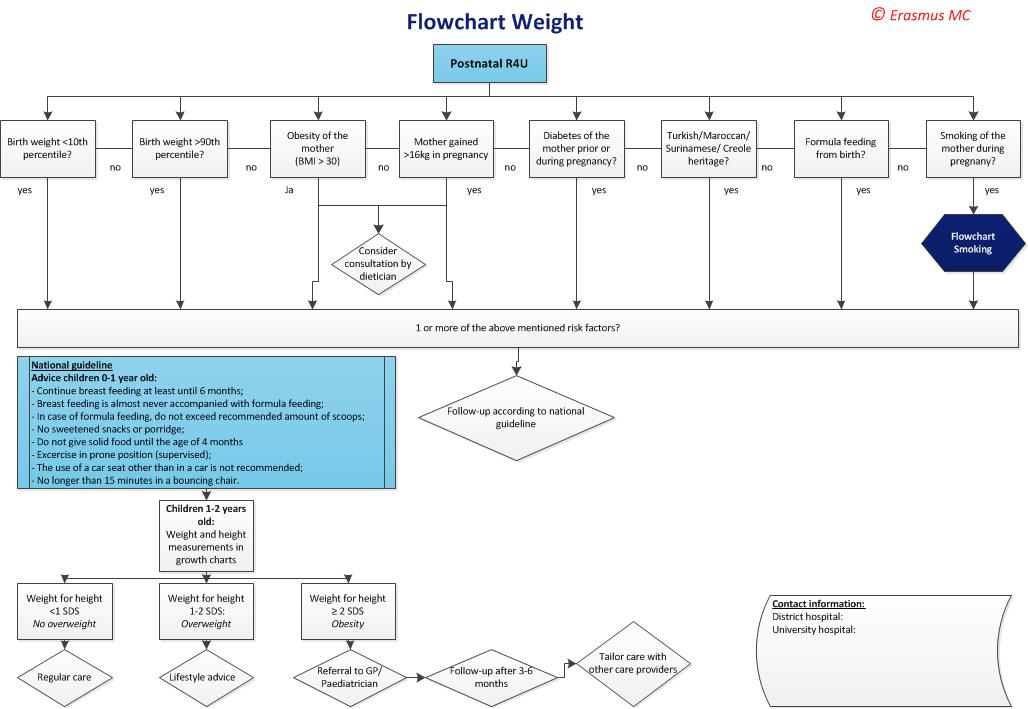

Supplement: S3 File — (DOCX) [file pone.0217261.s003.docx]
