# Supplementary material for: Reducing growth and developmental problems in children: Development of an innovative postnatal risk assessment
Source: PLoS One. 2019 Jun 5;14(6):e0217261. doi: 10.1371/journal.pone.0217261 (PMC6550373; doi:10.1371/journal.pone.0217261)
Supplement: S4 File — (DOCX) [file pone.0217261.s004.docx]

Question route focus group interview HP4All-2; JGZ project

Duration: a maximum of 1.5 hours

Introduction (5 min)

Introduction: introduction round participants, (nameplates and attendance list available).

I want to start by saying that I am glad you all want to participate in this focus group meeting. This meeting is one out of three.

We would like to introduce ourselves. I am Minke van Minde, physician-researcher. I work as a researcher for the project Healthy Pregnancy 4 All-2 (HP4All-2) of the Erasmus Medical Center for the research 'Risk factors that influence the growth and development of children up to 2 years of age'. This is Marlou de Kroon, senior researcher and my copromotor and this is Hiske Ernst, project manager of the JGZ and the maternity care project of HP4All-2.

We have brought one / two lady (s), namely ... ...

They will make notes during the focus group meeting.

Explanation of the project

I have sent you an e-mail with a brief explanation of the project. I will summarize briefly. <see presentation>. This research is being carried out on behalf of the Ministry of Health, Welfare and Sport. The research offers the opportunity to detect children with the risk of growth and development problems early and to offer them and their parents adequate care.

The aim of this PhD research is to show that early postnatal identification of children with risk factors and the use of care pathways reduces growth and development problems later in life. Probably attempted afterwards In the case of a positive result of the instrument, NCJ and VWS will be advised to implement the developed risk instrument nationally at the Youth Health Care on the basis of scientific outcomes.

Why are we here?

At this moment we are together with you to discuss which risk factors influence the growth and development of the young child. For practical reasons we limit ourselves to growth and development during the first two years of life.

Your insight and knowledge are important here: after all, you are the expert. We are curious about your experience and opinion.

I will soon discuss the risk factors that have been shown in scientific research to influence growth and development. We found these risk factors thanks to an extensive literature search. The data from the literature and the proceeds from the focus group meetings will be used to develop the postnatal risk instrument.

I would like to emphasize that no answer you give is right or wrong, it concerns your opinion and the arguments that you have in your opinion. Everything that is said during this meeting is confidential and will be processed anonymously.

I would like to use the voice recorder to record the interview so that I can work out the data afterwards. I guarantee that outside this group and the research group no one will have access to this recording.

Since we have a tight timetable and we would like to have all parts adequately covered, I took a call. When the time has elapsed for the relevant item, I will ring the bell to indicate that it is time for the next item.

All in all, the meeting will last about one and a half hours.

Do you have any questions?

On the screen I now show the following:

The results that we want to investigate are listed horizontally. To the left of this you will see the various risk factors that have been drawn up on the basis of literature research and the expectations of the researchers. Red indicates that the risk factor increases the chance of a less favorable outcome (such as overweight); green indicates that the factor reduces the chance of a less desirable outcome and that in fact there is a protective effect (such as breastfeeding) and light red indicates an expected effect that we have not yet been able to demonstrate with literature research. From this and possibly about other unknown to us? risk factors we would like to hear your opinion.

Outcomes

The outcomes we discuss are:

- Overweight / obesity

- Catch-up growth

- Motor development

- Speech / language development

- Cognitive development

- Psychosocial development

- Child abuse

The categories below which we have divided the risk factors are:

- General factors

- Social factors

- Lifestyle factors

- Medical factors

- Obstetric factors in the mother

- Obstetric factors in the child

1. Category general (min)

The literature shows that the following general factors are relevant for the growth and development of the child: height of father, siblings and maternal age.

A: Which risk factors of this list do you find relevant and to what extent?

B: What arguments do you have for this?

'When giving reasons, you can think of the seriousness of the disorder, the frequency, the treatability, possible follow-up actions, the effect of the follow-up actions on the individual and population level, or sensitivity / specificity'.

C: An argument that has not yet been mentioned is the following ... what do you think of this argument?

D: Which risk factors do you miss in this list? What arguments do you have for this?

E: Which risk factors do you think should be included in a risk assessment instrument (the postnatal R4U), in view of the related outcomes, catch-up growth, overweight, motor development, etc ...

F: Summarize points.

2. Social category (min)

The literature shows that the following socially related factors are important for the growth and development of the child: SES, ethnicity, maternal mental retardation, single parent and maternal high workload.

A: Which risk factors of this list do you find relevant and to what extent?

B: What arguments do you have for this?

'When giving reasons, you can think of the seriousness of the disorder, the frequency, the treatability, possible follow-up actions, the effect of the follow-up actions on the individual and population level, or sensitivity / specificity'.

C: An argument that has not yet been mentioned is the following ... what do you think of this argument?

D: Which risk factors do you miss in this list? What arguments do you have for this?

E: Which risk factors do you think should be included in a risk assessment instrument (the postnatal R4U), in view of the related outcomes, catch-up growth, overweight, motor development, etc ...

F: Summarize points.

3. Category lifestyle (min)

The literature shows that the following lifestyle factors are important for the growth and development of the child: smoking, obesity, overweight, underweight, alcohol, drugs, pets and humidity in the home.

A: Which risk factors of this list do you find relevant and to what extent?

B: What arguments do you have for this?

'When giving reasons, you can think of the seriousness of the disorder, the frequency, the treatability, possible follow-up actions, the effect of the follow-up actions on the individual and population level, or sensitivity / specificity'.

C: An argument that has not yet been mentioned is the following ... what do you think of this argument?

D: Which risk factors do you miss in this list? What arguments do you have for this?

E: Which risk factors do you think should be included in a risk assessment instrument (the postnatal R4U), in view of the related outcomes, catch-up growth, overweight, motor development, etc ...

F: Summarize points.

4. Medical category (min)

The literature shows that the following medically related factors are important for the growth and development of the child: bulimia nervosa, postnatal depression, depression during pregnancy, depression / anxiety, other mental disorder, paternal psychological pathology, diabetes mellitus, hypothyroidism, and certain medication.

A: Which risk factors of this list do you find relevant and to what extent?

B: What arguments do you have for this?

'When giving reasons, you can think of the seriousness of the disorder, the frequency, the treatability, possible follow-up actions, the effect of the follow-up actions on the individual and population level, or sensitivity / specificity'.

C: An argument that has not yet been mentioned is the following ... what do you think of this argument?

D: Which risk factors do you miss in this list? What arguments do you have for this?

E: Which risk factors do you think should be included in a risk assessment instrument (the postnatal R4U), in view of the related outcomes, catch-up growth, overweight, motor development, etc ...

F: Summarize points.

4. Category obstetric mother (min)

The literature shows that the following mother dependent obstetric related factors are important for the growth and development of the child: weight gain pregnancy, diabetes gravidarum, type of nutrition, parity, assisted reproduction, unwanted pregnancy, vitamin B 12 or folic acid deficiency.

A: Which risk factors of this list do you find relevant and to what extent?

B: What arguments do you have for this?

'When giving reasons, you can think of the seriousness of the disorder, the frequency, the treatability, possible follow-up actions, the effect of the follow-up actions on the individual and population level, or sensitivity / specificity'.

C: An argument that has not yet been mentioned is the following ... what do you think of this argument?

D: Which risk factors do you miss in this list? What arguments do you have for this?

E: Which risk factors do you think should be included in a risk assessment instrument (the postnatal R4U), in view of the related outcomes, catch-up growth, overweight, motor development, etc ...

F: Summarize points.

5. Category obstetric child (min)

The literature shows that the following child-dependent obstetric related factors are important for the growth and development of the child: macrosomy, IUGR, dysmature, premature, amenorrhea 37-38 weeks, vaginal breech delivery, congenital abnormality, low APGAR score and multiple births.

A: Which risk factors of this list do you find relevant and to what extent?

B: What arguments do you have for this?

'When giving reasons, you can think of the seriousness of the disorder, the frequency, the treatability, possible follow-up actions, the effect of the follow-up actions on the individual and population level, or sensitivity / specificity'.

C: An argument that has not yet been mentioned is the following ... what do you think of this argument?

D: Which risk factors do you miss in this list? What arguments do you have for this?

E: Which risk factors do you think should be included in a risk assessment instrument (the postnatal R4U), in view of the related outcomes, catch-up growth, overweight, motor development, etc ...

F: Summarize points.

6. Missing risk factors (10 min)

A. What risk factors are you missing?

B. A risk factor that has not been mentioned is ......

An argument for this is .....

What do you think of this argument? what argument?

C. Does this risk factor still influence other risk factors?

D. A competence that has not been mentioned is ......

E. Summarize points

6. Implementation of the risk instrument (10 min)

How can we best implement this risk instrument?

Counseling takes place before the risk assessment instrument is used. In this, the relevant JGZ employee, usually the youth nurse, explains the research to the parents and requests permission to purchase the instrument.

A. How long can the counseling last?

B. An argument that has not yet been mentioned is the following ......

what do you think of this argument?

C. How stressful is the decrease of the postnatal R4U for the child? What does this depend on?

How stressful can the purchase be for parents and child?

D. An argument that has not yet been mentioned is the following ......

what do you think of this argument?

E. Summarize points

SUMMED whole conversation

Exit (5 min)

These were all questions that I wanted to ask you.

Do you have any questions or comments? Or is there anything else you would like to give us as advice? We will process everything that has been said during this meeting and see what opinions and arguments have emerged. Based on the focus group meetings and the literature, we will develop and apply the postnatal R4U in the research. If you have indicated that you want to be kept informed of the developments of the research, you will receive a message.

(Email addresses are already in possession of HP4All-2, see log focus group).
